# Polydopamine Nanoparticles as Label-Free Contrast Agents in Photoacoustic Imaging *In Vitro* and *In Vivo*

**DOI:** 10.1021/acsaom.5c00637

**Published:** 2026-03-23

**Authors:** Matteo Battaglini, Paolo Armanetti, Alessio Carmignani, Claudia Catarinicchia, Valentina Naef, Margherita Montorsi, Marie Celine Lefevre, Claudio Canale, Davide Odino, Luca Menichetti, Filippo Maria Santorelli, Gianni Ciofani

**Affiliations:** 1 Istituto Italiano di Tecnologia, Smart Bio-Interfaces, Viale Rinaldo Piaggio 34, Pontedera 56025, Italy; 2 National Research Council, Institute of Clinical Physiology, Via Giuseppe Moruzzi 1, Pisa 56124, Italy; 3 Unit of Neurobiology, 9357 IRCCS Stella Maris Foundation, Via dei Giacinti 2, Calambrone (Pisa) 56128, Italy; 4 Molecular Medicine and Neurogenetics, 9357 IRCCS Stella Maris Foundation, Via dei Giacinti 2, Calambrone (Pisa) 56128, Italy; 5 National Research Council, Institute for the Chemical and Physical Processes, Largo Pontecorvo 3, Pisa 56127, Italy; 6 Department of Physics, 9302 University of Genova, Via Dodecaneso 33, Genova 16146, Italy; 7 Scuola Superiore Sant’Anna, Health Sciences Interdisciplinary Center, Piazza Martiri della Libertà 33, Pisa 56127, Italy

**Keywords:** photoacoustic imaging, contrast agents, theranostic nanomaterials, polydopamine nanoparticles, alternative in vivo models

## Abstract

Polydopamine nanoparticles (PDNPs) are a class of nanomaterials formed by the self-polymerization of dopamine. They exhibit high biocompatibility, biodegradability, antioxidant properties, and ease of functionalization and can serve as photothermal agents when exposed to near-infrared (NIR) light. Another notable feature of PDNPs is their potential to act as contrast agents in photoacoustic imaging (PAI). In this technique, light absorption by endogenous chromophores or nanostructures induces thermal expansion, which generates sound waves that can be exploited to create images. Although PDNPs have shown promise as PAI contrast agents, their capabilities remain underexplored and insufficiently characterized in biological systems. This study presents the first comprehensive evaluation of PDNPs as PAI contrast agents. We investigated PDNPs of various sizes (∼150–1000 nm) and assessed their photoacoustic performance in diverse environments, including aqueous dispersions, *ex vivo* tissues, U87 cancer cell spheroids, fertilized quail eggs, and zebrafish embryos. Additionally, experimental results supported the development of a computational model to predict PDNP photoacoustic properties. Overall, this work highlights the significant, yet largely unexplored, potential of PDNPs as label-free PAI contrast agents, contributing to their future exploitation in clinical imaging.

## Graphical abstract

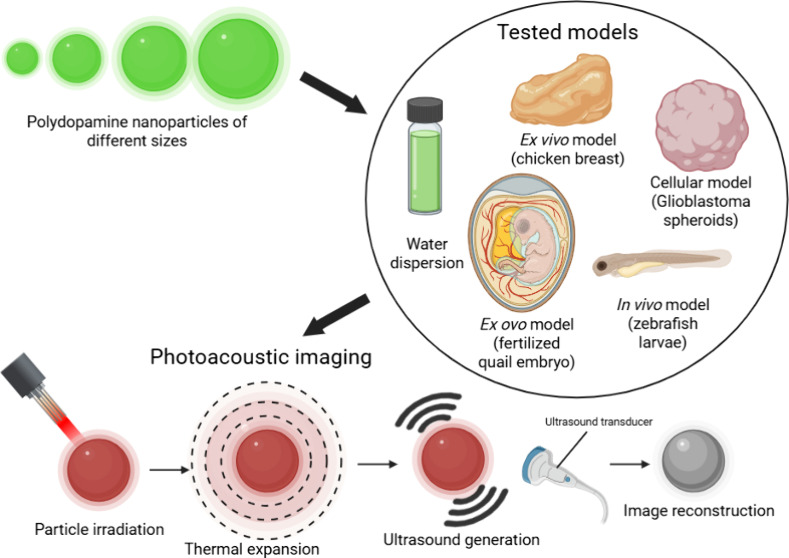

## 1 Introduction

Polydopamine nanoparticles (PDNPs) are nanostructures derived from the self-polymerization of dopamine.^1^ PDNPs have been extensively exploited for biomedical applications because of their remarkable features, which include high biocompatibility and biodegradability,^2^ antioxidant properties,^3^ the ability to be easily functionalized with a large variety of molecules^4^ and the capacity to act as photothermal conversion agents when irradiated with a near-infrared (NIR) laser source.^5^ Some of the PDNP potential applications include the treatment of neurological disorders,^2^ the treatment of various forms of cancer, including liver,^6^ colorectal,^7^ and brain^8^ cancer, wound healing,^9^ and as countermeasure against liver steatosis.^10^

Another property of PDNPs is their capacity to act as contrast agents in photoacoustic imaging (PAI),^11^ a hybrid imaging technique combining illumination and ultrasound (US) detection. PAI is based on the irradiation of samples with a nanosecond pulsed laser (pulse duration <10 ns). The irradiation is absorbed by the molecules of the samples, which generate heat.^12–14^ The heat causes a thermoelastic expansion, generating acoustic waves that can be detected by exploiting ultrasonic transducers to produce images.^12–14^ The main advantage of PAI is that the acoustic signal can propagate within tissues without significant attenuation, conversely to light-based signals.^12–14^ PAI has several advantages over other imaging techniques commonly exploited in clinics: More in detail, PAI possesses better molecular sensitivity than fluorescence imaging and better spatial resolution than ultrasound imaging, does not employ ionizing radiation like computed tomography (CT) or positron emission tomography (PET), and is faster than other techniques like magnetic resonance imaging (MRI).^15–18^

PAI can exploit the light absorption properties of endogenous chromophores such as lipids, melanin, hemoglobin, and deoxyhemoglobin to visualize biological structures;^19–25^ however, endogenous chromophores are of limited usefulness because of the low intensity of their PA signals, unsuitable to image nontransparent structures.^19^ To overcome the limitations posed by endogenous chromophores, an exogenous contrast agent can be exploited to enhance PAI signal. Some of the most used PAI contrast agents include fluorescent dyes like indocyanine green (ICG), methylene blue (MB), and Evans blue (EB),^26–28^ or light-absorbing nanostructures like gold nanomaterials,^29,30^ carbon nanostructures,^31^ and molybdenum oxide (MoO_3_) nanoparticles.^32^ Both classes of contrast agents have advantages and limitations that hinder their wide exploitation for biomedical applications. Organic dyes are relatively small and highly biocompatible; however, they suffer from low stability, being easily photobleached by the laser exploited in PAI, and they can act as PA contrast agents only in a restricted wavelength window. On the other hand, inorganic nanostructures are more photostable, can be engineered to absorb light in a broader range of wavelengths, and can generate a higher PA signals; however, they are limited by their potential toxicity and lack of biodegradability.^19^ Therefore, there is a strong need to develop PAI contrast agents that combine the PA properties of nanostructures with the high biocompatibility of organic dyes.

PDNPs could represent an ideal candidate in this regard. The PA properties of polydopamine nanostructures have been mentioned in a few works in the literature where they have been exploited as PAI contrast agents in cervical cancer,^33^ endometriosis lesions,^11^ and breast cancer.^34^ PDNP's ability to act as a PAI contrast agent is due to their ability to act as photothermal conversion agents when irradiated with a light source at a proper wavelength, and to their chemical structure analogous to melanin, which, as we previously mentioned, is a well-known endogenous PA chromophore.^1^ Despite their potential, the exploitation of PDNPs as a PAI contrast agent is still severely underexplored, as shown by the minimal amount of work exploiting this property.

The objective of this work is to provide the first complete analysis and characterization of the PA properties of PDNPs in relevant biological environments. To achieve this goal, we tested the PA properties of PDNPs of different sizes (from approximately 150 up to 1000 nm). After a morphological and mechanical characterization, the PA properties of PDNPs were assessed in water, in an *ex vivo* model (chicken breast), in a cellular model (U87 spheroids), in an *ex ovo* model (fertilized quail eggs), and in an *in vivo* model (Zebrafish embryos, *Danio rerio*). The data collected in our tests were then exploited to develop a descriptive and predictive computational model of the PA properties of PDNPs, to show how the physical properties of the nanostructures can affect their performance as a PAI contrast agent.

While polydopamine nanoparticles have been explored as PAI contrast agents in several earlier studies, these works typically focused on a single nanoparticle size or on formulations below 200 nm primarily designed for drug delivery.^35^ Conversely, the present study employs a systematically engineered library of eight narrowly distributed PDNP sizes spanning nearly one order of magnitude (145–957 nm) and provides the first comprehensive, size-resolved evaluation of their photoacoustic performance across multiple biological complexity levels. Moreover, we combine these experiments with new colloidal stability measurements, *in vitro* and *in vivo* biocompatibility assessments of the exact batches used, and a dual-scale computational model to mechanistically link PDNP size, thermoelastic behavior, and PA signal generation. This integrated approach allows us to investigate aspects of PDNP behavior that have not been addressed before.

## 2 Materials and Methods

### 2.1 Nanoparticle Synthesis

PDNPs were prepared by exploiting an adaptation of the Stöber reaction, as previously described.^5,36^ Briefly, 90 mL of Milli-Q water, 40 mL of ethanol, and different volumes of ammonium hydroxide (Carlo Erba) were mixed under mild stirring at room temperature for 30 min. Afterward, 0.5 g of dopamine hydrochloride (Sigma) dissolved in 10 mL of Milli-Q water was added to the reaction mix. To modulate the PDNP diameter, eight different molar ratios of ammonium hydroxide/dopamine hydrochloride were used (23.20, 17.40, 14.50, 11.60, 8.70, 5.80, 4.35, and 2.9). The reaction was kept stirring in the dark overnight; then, the mix was diluted 1:1 with acetone and centrifuged at 8960 *g* for 30 min at 4 °C. The obtained PDNP pellet was collected and resuspended in Milli-Q water for three subsequent washing steps by centrifugation at 16,602 *g* at 4 °C for 30 min. The PDNP concentration was quantified by weighing selected freeze-dried aliquots.

### 2.2 Scanning and Transmission Electron Microscopy

The morphology and diameter of PDNPs were determined through scanning electron microscopy (SEM) analysis and by exploiting Gwydion software. For each of the eight PDNP groups, 10 μL of a 100 μg/mL water suspension was placed on a silicon substrate and let dry under a chemical hood. After gold sputtering using a Quorum SC7620 mini–Gold Sputter Coater (15 mA for 30 s), images were acquired using a dual-beam SEM system (Helios NanoLab 600i FIB/SEM, FEI).

For transmission electron microscopy (TEM) analysis, PDNP dispersions were sonicated (20 min) and drop-cast on a Cu TEM grid with a 200 hexagonal mesh. TEM images were obtained with a JEOL JEM 1011 electron microscope and recorded with a 2 MP charge-coupled device camera (Orius Gatan). Before analysis, PDNPs were stained with 1% uranyl acetate in Milli-Q water for 1 min.

### 2.3 Atomic Force Microscopy Analysis

Samples for AFM imaging experiments were prepared by diluting nanoparticles in Milli-Q water to a final concentration of approximately 30 μg/mL. Aliquots of 50 μL were deposited on a freshly cleaved mica substrate. After 20 min of incubation, samples were rinsed with Milli-Q water and dried under a mild vacuum. A JPK NanoWizard IV microscope (Bruker) was exploited, operating in tapping mode in air using single-beam uncoated silicon cantilevers (TESPA-V2, Bruker) with a nominal spring constant of 42 N/m, a resonance frequency in the air between 320 and 340 kHz, and a typical tip radius of curvature of 10 nm. Images with a scan size of 10 μm × 10 and 4 μm × 4 μm (512 pixels × 512 pixels) were acquired.

The elastic modulus of PDNPs was analyzed in quantitative imaging mode (QI, JPK Instruments) in a liquid environment using the NanoWizard IV microscope (Bruker). In this mode, topographic images and force–distance curve maps were obtained simultaneously.^37^ TESPA-V2 (Bruker) was also used for nanoindentation experiments. One of the main disadvantages of nanoparticles is their low adhesion to the mica substrate; to overcome this limitation, PDNPs were covalently attached to mica substrates chemically functionalized with APTES/glutaraldehyde. The freshly cleaved mica substrates were silanized in 5 μL of APTES (vapor phase silanization) for 5 min and thereafter further functionalized with a 2.5% glutaraldehyde solution; after 20 min of incubation, they were rinsed with Milli-Q water. Subsequently, 50 μL of PDNPs 0.5 mg/mL in Tris buffer (10 mM, pH 8.5) was deposited on the functionalized mica substrate and incubated for 1 h. The surfaces were then gently rinsed with Milli-Q water. Finally, Tris buffer (10 mM, pH 7.4) was added for AFM measurements. QI images were collected with scan sizes of 2.5 μm × 2.5 μm (resolution 128 pixels × 128 pixels) and 5 μm × 5 μm (resolution 256 pixels × 256 pixels), with a set point force of 35 nN and with an indentation speed of 50 μm/s. Before each experiment, the sensitivity of the cantilever deflection was determined by acquiring a force–distance curve on a mica disk working in Tris buffer (10 mM, pH 7.4), and the spring constant was evaluated by the thermal noise method^38^ implemented in the JPK Calibration Manager software. Young’s modulus associated with each pixel was extracted by processing the experimental force–distance curves using JPK Data Processing software (Bruker); then, the Hertz–Sneddon model, valid for spherical indenters, was fitted to the approach curve.

Operating on small spherical samples, the geometry of the contact between the tip (assumed as a spherical object with a diameter of 10 nm) and the particle under investigation is changing from the particle center to the particle periphery. Following the method proposed by Zmerli et al.,^39^ force–distance curves were isolated and analyzed at the center of individual NPs. This analysis allows Young’s modulus values at the edges of the nanoparticles to be neglected, which are lower due to geometric artifacts originated by the contact between the tip and the edges of the nanoparticles, in agreement with a previous work.^37^

### 2.4 Dynamic Light Scattering and UV–vis-NIR Spectroscopy

PDNP dispersions were characterized through dynamic light scattering (DLS) measurements in terms of average hydrodynamic diameter and average surface zeta potential (ζ-pot) using a Malvern Zetasizer Nano ZS90. Hydrodynamic diameter measurements were performed on 100 μg/mL PDNPs in Milli-Q water using polystyrene cuvettes, while ζ-pot measurements were performed on 100 μg/mL PDNPs in Milli-Q water placed in folded capillary cells (Malvern).

To evaluate colloidal stability under biologically relevant conditions, PDNPs of all eight diameters were incubated at a concentration of 100 μg/mL in complete cell culture medium. The medium consisted of high-glucose Dulbecco’s modified Eagle’s medium (DMEM, Sigma-Aldrich) supplemented with 10% heat-inactivated fetal bovine serum (FBS, Gibco), 1% l-glutamine (stock 200 mM, Gibco), 1% sodium pyruvate (stock 100 mM, Gibco), and 1% penicillin–streptomycin (100 IU/mL penicillin and 100 μg/mL streptomycin, Gibco). Nanoparticle suspensions were incubated at 37 °C under standard cell culture conditions. At defined time points (1, 24, and 48 h), the particles were analyzed by DLS to determine changes in hydrodynamic diameter and polydispersity index (PDI). Measurements were performed without dilution to preserve the nanoparticle–protein interactions occurring in the medium. Each measurement was conducted in triplicate, and results are reported as mean ± standard deviation.

Absorbance spectra of aqueous PDNP dispersions (100 μg/mL) were recorded in the 680–970 nm range using a PerkinElmer UV/vis spectrophotometer (Lambda 45). Measurements were performed directly in water, without additional treatment, to evaluate the intrinsic near-infrared optical absorption properties of nanoparticles with different nominal diameters.

### 2.5 Biocompatibility Studies

The biocompatibility of the PDNPs used in this work was analyzed on U87 glioblastoma cells (ATCC) using a Live/Dead assay, and in zebrafish embryos.

For the Live/Dead assay, U87 glioblastoma cells were seeded in 48-well plates (Corning) at a density of 10,000 cells/cm^2^ and allowed to adhere for 24 h under standard culture conditions (cells were grown using the medium described in Section 2.4). Cells were subsequently exposed to PDNPs of eight nominal diameters (ranging from 145 to 957 nm) at two concentrations (100 and 200 μg/mL). Treatments were carried out for 24 and 72 h. Following incubation, cells were washed with Dulbecco’s phosphate-buffered saline (DPBS) and incubated for 20 min in phenol red-free culture medium supplemented with 5 μg/mL Hoechst 33342 (Invitrogen), 4 μM ethidium homodimer-1, and 2 μM calcein-AM (Thermo Fisher Scientific). After staining, cells were rinsed again with DPBS and immediately imaged using a fluorescence microscope equipped with a 10× objective. Cell viability was determined by quantifying the proportion of live (calcein-positive) and dead (ethidium homodimer-1-positive) cells under each experimental condition.

To define a safe and biologically relevant dose for zebrafish *in vivo* experiments, a dose-finding strategy was adopted that accounted for larval size, biodistribution, and potential differences in sensitivity relative to *in vitro* cellular models. Zebrafish larvae at 48 hpf were injected with ∼2 nL of nanoparticle suspension at four different concentrations (50, 100, 200, and 300 μg/mL), corresponding to absolute doses of approximately 0.1, 0.2, 0.4, and 0.6 ng *per* larva, respectively. Only morphologically normal larvae that had spontaneously exited the yolk sac were selected for injection, to avoid potential artifacts associated with enzymatic or mechanical dechorionation. All experiments were performed separately for each nanoparticle size tested (145, 522, and 957 nm). Larval survival was assessed at 72 hpf (24 h postinjection), while morphological parameters (eye size and body length) and locomotor activity (distance traveled and velocity), commonly used as indicators of developmental and functional toxicity in zebrafish models, were evaluated at 120 hpf.^40^ Based on the dose-dependent effects observed, 50 μg/mL was selected for all subsequent experiments. The absolute mass of nanoparticles injected *per* embryo is approximately 0.1 ng at the selected concentration of 50 μg/mL (∼2 nL per embryo).

### 2.6 PAI in Water Dispersion

PA evaluations were performed using the multimodal imaging platform Vevo LAZR-X from FUJIFILM VisualSonics Inc. The PA properties of PDNPs were first evaluated in a custom-made test-object phantom, which was validated in a previous work,^41^ and made of a polypropylene (PP) box containing coplanar polyethylene (PE) micrometric tubes. These tubes have an internal diameter of 0.58 mm and an external diameter of 0.99 mm and were filled with PDNPs of eight different nominal diameters (145, 186, 204, 299, 426, 522, 710, and 957 nm) in water at 0.2 mg/mL. The PA multispectral analysis was conducted within the 680 to 970 nm wavelength range to identify their specific fingerprint. Photostability was evaluated by keeping the PDNPs under prolonged laser illumination at a fixed wavelength (705 nm). Lastly, the contrast-to-noise ratio (CNR), the signal-to-noise ratio (SNR), and the percent coefficient of variation (%CV) were calculated according to, respectively, eqs 1, 2, and 3:

$$CNR=\frac{S-b}{\sqrt{\sigma_{S}^{2}-\sigma_{b}^{2}}}$$ 1

$$SNR=\frac{S}{\sigma_{S}}$$ 2

$$\%CV=100\cdot \frac{\sigma_{S}}{S}$$ 3

where *S* is the acquired PA signal, *b* is the background, and σ is the standard deviation.

The PA signals were studied in the tube section of the PA images by drawing a region of interest (ROI) of the same dimensions. Then, they were processed and analyzed using VEVOLAB software from FUJIFILM Visualsonics. As a positive control, 200 μg/mL of ICG (Pulsion) was also imaged through PAI.

All measurements were performed using a Yag pulsed nanosecond laser with an optical parametric oscillator (OPO), with a pulse duration of ∼8 ns and an irradiation energy of 36 mJ at 808 nm.

### 2.7 PAI *Ex Viv*o, *In Vitro, Ex Ovo,* and *In Vivo*

The concentrations of PDNPs used in this study were selected to ensure a robust and quantifiable PA signal while remaining within biocompatible ranges. Previous reports from our group demonstrated that PDNPs synthesized with the same protocol are nontoxic to neuronal, endothelial, and fibroblast cells at doses up to 200 μg/mL.^2,5,3,42^ In the zebrafish model, doses were adjusted proportionally to body volume and optical requirements, in line with prior studies confirming good tolerance of melanin-like nanoparticles *in vivo.* ^43^ No abnormal behavior, morphology, or mortality was observed at the tested doses, consistent with the well-established biocompatibility of PDNPs.^1^

A bolus of 50 μL of 0.2 mg/mL PDNPs of eight different nominal diameters (145, 186, 204, 299, 426, 522, 710, and 957 nm) was injected into an *ex vivo* chicken breast sample to simulate a biological environment. The PA signals were studied by drawing an ROI around the injection site of the PA images, which were processed and analyzed by the VEVOLAB software to perform PA multispectral analysis, photostability assessment, and evaluation image quality parameters (SNR, CNR, %CV; as previously reported).

The possibility of exploiting PDNPs for PAI in cellular models was tested using spheroids derived from U87 cells. Briefly, 100 μL of agarose (Sigma) was deposited on the bottom of 96-well plates (Corning). After cooling, 10,000 U87 cells were seeded in the previously prepared agarose-coated 96-well plate, centrifuged at 300*g*, and allowed to grow for 5 days with a single medium change after 3 days of culture. Afterward, the obtained spheroids were treated with 200 μg/mL of PDNPs of different sizes (186, 204, 299, 426, 522, 710, and 957 nm in diameter) for 72 h. The treated spheroids were washed twice in Dulbecco’s phosphate-buffered saline (DPBS) and fixed in PFA 4% in DPBS at 4 °C for 20 min. After fixation, the spheroids were washed with DPBS and imaged in an agarose structure of 0.5% w/v: The setup geometry consisted of an agarose hollow cylinder where the spheroids were placed; then, the hole was filled with physiological solution and covered with echographic gel for PA probe coupling. The PA multispectral analysis was carried out within the 680–970 nm range, while the photostability was assessed under prolonged laser illumination at 700 nm. CNR, SNR, and %CV parameters were evaluated for each sample.

Fertilized eggs of Japanese quail (*Coturnix japonica*) were purchased from Japocaille and incubated for 4 days at 37 °C and 57% humidity in an egg incubator with a 360° rotating roller for automatic horizontal turning every 2 h. Embryos were gently extracted from the shell, transferred into a Petri dish to perform shell-less culture, and returned to the egg incubator for 3 days. 50 μL of PDNPs at 2 mg/mL (of three different nominal diameters: 145, 522, and 957 nm) was injected into the chorioallantoic membrane with a 26G syringe needle. The embryos were then fixed with 4% PFA for 24 h, rinsed four times in PBS for 20 min, and stored in 70% ethanol until analysis.

The fixed embryos were embedded in agarose (Sigma) (1%) to keep the sample geometry stable and then coupled with echographic gel to the PA probe. Three-dimensional (3D) distribution of the PA signal within the fixed embryo was acquired using the multiwavelength mode. In detail, sample volumes were scanned at a step size of 150 μm, and for each slice, the PA signal was retrieved at four different wavelengths (680, 700, 924, and 970 nm). The 3D PA acquisitions were processed to separate the mixed spectral signals of PDNPs, endogenous responsive molecules (oxygenated hemoglobin, deoxygenated hemoglobin), and noise, into their contributions within the samples. The characteristic spectra of each component against which this discrimination was performed were obtained as follows: for oxygenated and deoxygenated hemoglobin, default spectra provided by the software were used; for PDNPs and noise, spectra acquired from corresponding regions of interest were exploited (regions with the presence of air bubbles for noise, and phantom test tubes for PDNPs). The PA signal derived from PDNPs of three different diameters measured at various wavelengths was normalized by the maximum value of each spectrum.

Adult wild-type AB zebrafish strains were maintained in a recirculating water system at a temperature between 26.0 and 28.5 °C, under a 14 h light/10 h dark cycle. Zebrafish eggs and embryos were collected and raised at 28.5 °C in E3 embryo water using established procedures.^43^ All experiments conducted in this study comply with European Community standards for the use of animals in experimentation, under the supervision of the Institutional Animal Care and Use Committee of the University of Pisa (Pisa, Italy) and adhere to the 3R principles.^44^ Zebrafish embryos were anesthetized by immersion in E3 embryo water supplemented with 0.02% tricaine (Sigma-Aldrich) and placed on a plate containing 2% low melting-agarose (Sigma). PDNPs (50 μg/mL in water, ∼2 nL) of three different nominal diameters (145, 522, and 957 nm) were injected into the yolk sac of 48 hpf (hours post fertilization)-old wild-type AB larvae.^45^ At least three independent injection experiments were performed with spawns from different founder fish, to control for batch effect. Larvae were fixed at 72 or 120 hpf with 4% formaldehyde (FA) in PBS. Each fixed larva was placed in a single well of a 96-well plate (Corning) and embedded in a 370 μL volume of agarose (Sigma). After cooling, each cylindrical-shaped sample was imaged as previously reported. PA multispectral analysis was performed in the 680–970 nm wavelength range, while photostability was evaluated under prolonged laser illumination at 700 nm. The CNR, SNR, and %CV parameters were also assessed for each sample. Concerning the *in vivo* analysis, all the measurements exploited energies below 25 mJ/cm^2^, in line with safety requirements for *in vivo* applications of PAI.^46^

The physical parameters of the laser, namely, pulse energy and repetition rate, were kept constant across all imaging models. These parameters correspond to the system’s default settings, which are preoptimized for biological tissue visualization. No tissue-specific optimization was performed, as the system is designed to deliver consistent and reliable photoacoustic imaging performance under these standard conditions. To ensure stable and reproducible operating values, prior to each PA imaging session, a laser calibration procedure is carried out, as recommended by the manufacturer.

### 2.8 Computational Model of PDNP PAI Imaging

The photoacoustic pressure in the 3D far-field model in COMSOL Multiphysics FEM software was calculated by exploiting the “Thermoviscous Acoustic Module” as described by Handte et al.^47^ The linearized governing equations exploited by the software to model the phenomena are the continuity equation (eq 4), the momentum equations (eqs 5 and 6), the energy conservation equation (eq 7), and the linearized equation of state, which connects variations in pressure, temperature, and density in a fluid (eq 8):

$$i\omega \rho_{t}\nabla \cdot \left(\rho_{0}\cdot u_{t}\right)=0$$ 4

$$i\omega \rho_{0}\cdot u_{t}=\nabla \cdot \sigma$$ 5

$$\sigma =(-p_{t}I+\mu \left(\nabla u_{t}+{\left(\nabla u_{t}\right)}^{T}\right)-\left(\frac{2}{3}\mu -\mu_{B}\right)\left(\nabla \cdot u_{t}\right)I$$ 6

$$\rho_{0}C_{P}\left(i\omega T_{t}+u_{t}\cdot \nabla T_{0}\right)-\alpha_{p}T_{0}\left(i\omega p_{t}+u_{t}\cdot \nabla p_{0}\right)=\nabla \cdot \left(k\nabla T_{t}\right)+Q_{abs}$$ 7

$$\rho_{t}=\rho_{0}\left(\beta_{T}p_{t}-\alpha_{P}T_{t}\right)$$ 8

where ρ_0_ [kg/m^3^], *T*_0_ [K], *u*_0_ = (*u*_0*x*_, *u*_0*y*_, *u*_0*z*_) [m s^–1^], and *p*_0_ [Pa] are the background mean values of density, temperature, acoustic velocity, and pressure, respectively.

Equations 9, 10, and 11 describe, respectively, the effective values of temperature, velocity, and pressure to be computationally solved by the model.

$$T_{t}=T_{0}+Te^{i\omega t}$$ 9

$$u_{t}=u_{0}+ue^{i\omega t}$$ 10

$$p_{t}=p_{0}+pe^{i\omega t}$$ 11

The physics interface solves the equations in the frequency domain, assuming all fields and sources to be harmonic. The harmonic variation of all fields and sources is given by *e* ^*i*ω*t*^. The subscript *t* indicates the time-harmonic evolution of the variables. In thermoviscous acoustics, the fluid is assumed to be quiescent at the beginning so that *u*_0_ = 0. The background pressure *p*_0_ is specified as the atmospheric one. The background temperature *T*_0_ is set at 309 K.

Equation 6 defines the stress tensor σ on a fluid element [N m^–2^], with *I* being the identity tensor and μ and μ_B_ [Pa s] the dynamic and bulk viscosities, respectively. In the energy conservation equation, *C*_P_ [J kg^–1^ K^–1^] represents the specific heat of polydopamine at constant pressure, *k* [W m^–1^ K^–1^] is its thermal conductivity, α_P_ [K^–1^] is the thermal expansion coefficient, and β is the isothermal compressibility [Pa^–1^].

The additional term *Q*_abs_ [W m^–3^] in the energy conservation equation denotes the heat absorbed by the system during NIR-laser stimulation: A separate multiphysics simulation was carried out exploiting the “Heat Transfer with Radiative Beam in Absorbing Media” multiphysics coupling to characterize this source term. This interface simulates heat transfer by conduction, convection, and radiation in absorbing media. The attenuation of an incident beam intensity within a semitransparent material due to absorption is characterized using the Beer–Lambert law. As the beam is absorbed, it releases energy, which serves as a heat source, according to eq 12:

$$Q_{abs}=\sum_{i}kI_{i}$$ 12

Here, *k* [m^–1^] represents the absorption coefficient and *I*_*i*_ is the intensity of the *i*th laser beam [W m^–2^], the solution of the Lambert–Beer equation.

The incident laser source was defined based on the experimental setup. Specifically, the laser peak energy was approximately 50 mJ, with a pulse duration τ_w_ < 10 ns; thus, the power was calculated by dividing the pulse energy by the pulse duration, obtaining *W*_LASER_= 50 MW. Knowing the surface area of the laser sources, the power density can be easily calculated; this boundary condition allows for the numerical solution of the absorbed heat, which is essential for the thermo-acoustic model. To assess the difference in the PA transduction capabilities among PDNPs of different sizes, parametric simulations were conducted by varying the absorption coefficient (based on our previous studies on photothermal conversion properties of PDNPs).^5^

The near-field model of photoacoustic signal from a single nanoparticle was exploited following the same approach described in the work from Hatef et al.^48^ The finite element (FE) method uses three COMSOL Multiphysics software modules to solve the fully coupled system of partial differential equations (PDEs). These equations include the transient thermal response of the NP and the surrounding medium to an incident laser source (as specified for the far-field model, eq 13):

$$\rho C_{P}\frac{\partial T}{\partial t}=\nabla k\nabla T+Q_{abs}\cdot f\left(t_{w}\right)$$ 13

ρ [kg m^–3^], *C*_P_ [J/kg K], and κ [W/m K] are the density, heat capacity, and thermal conductivity, respectively, while *T* is the temperature [K]. The function, *f*(τ_w_), represents the temporal shape of the incident laser pulse, defined by eq 14:

$$f\left(\tau_{W}\right)=\{\begin{array}{c} 10\leq t\leq \tau_{w}\\ 0t>\tau_{w} \end{array}$$ 14

The transient temperature rise of PDNPs has been incorporated in the structural mechanics model of the linear thermal expansion to evaluate the stress and strain tensor, according to Duhamel–Hooke’s law (eq 15):

$$s=C:\left(\epsilon -\alpha_{P}\left(T-T_{0}\right)\right)$$ 15

where *s* (Pa) and ϵ are the stress and strain tensors, α_P_ (K^–1^) is the thermal expansion tensor, and *T* and *T*_0_ are the current and reference temperatures [K]. *C* (Pa) is the fourth-order elasticity tensor associated with shear and bulk modulus.

To evaluate the PA pressure generation near the surface of the NPs, the structural displacement *d* of the nanoparticle surface due to expansion and relaxation is used as the boundary parameter of the transient acoustic simulation, according to eq 16:

$$n\left(\frac{1}{\rho }\nabla p_{t}\right)=-n\frac{\partial^{2}d}{\partial t^{2}}$$ 16

where *n* is the outward versor normal to the boundary, ρ is the density [kg/m^3^], *p*_t_ is the pressure [Pa], and *d* is the displacement [m]. Here, we introduce two concentric spherical domains, with the inner one representing the nanoparticle and the outer one representing the surrounding water, with a diameter *d*_out_ = 10 *d*_IN_. The PA signal is based on the function of the photothermal expansion of the inner domain of the nanoparticles. In our simulation, the absorbed heat due to NIR-laser interaction with PDNPs and the surrounding medium is used in heat transfer analysis to represent heat transfer analysis. The temperature rise in the PDNPs and surrounding medium is used in linear elastic thermal expansion calculations of the nanoparticle and structural mechanics analysis; eventually, the pressure wave generated by nanoparticle thermal expansion is exploited as the boundary coupling parameter between structural mechanics and acoustics simulations. The acoustic pressure was evaluated at a distance of 5000 nm from the nanoparticle surface, corresponding to approximately 10 times the particle diameter, in order to capture the local pressure field in the surrounding medium while keeping the computational load manageable, as previously adopted in related modeling studies.^47–51^

## 3 Results and Discussion

### 3.1 PDNP Characterization

PDNPs of different sizes were successfully obtained through a Stöber reaction and characterized in size, morphology, hydrodynamic diameter, and ζ-pot. By tuning the pH of the reaction, we successfully obtained PDNPs ranging from approximately 100 to 1000 nm with homogeneous morphology, as extensively described in previous works from our group.^5^

Images obtained with SEM and TEM are shown in Figure 1a and Figure 1b, respectively. We estimated the diameters of the various nanostructures from the multiple images to be 145 ± 13, 186 ± 15, 204 ± 18, 299 ± 31, 426 ± 45, 522 ± 70, 710 ± 57, and 957 ± 48 nm (these values are considered as the “nominal” diameters throughout all the manuscript), as already reported in Carmignani et al.^5,3^ The images also show how the nanostructures are spherical in shape and homogeneous in size. The measured hydrodynamic diameters shown in Figure S1 are 188 ± 2, 232 ± 5, 262 ± 2, 331 ± 4, 573 ± 30, 633 ± 30, 923 ± 90, and 1108 ± 60 nm; the ζ-pot values of the nanostructures were also measured as shown in Figure S2 and were equal to −37 ± 1, −40 ± 1, −49 ± 1, −47 ± 1, −44 ± 1, −47 ± 1, −51 ± 1, and −41 ± 1 mV.

**Figure 1:**
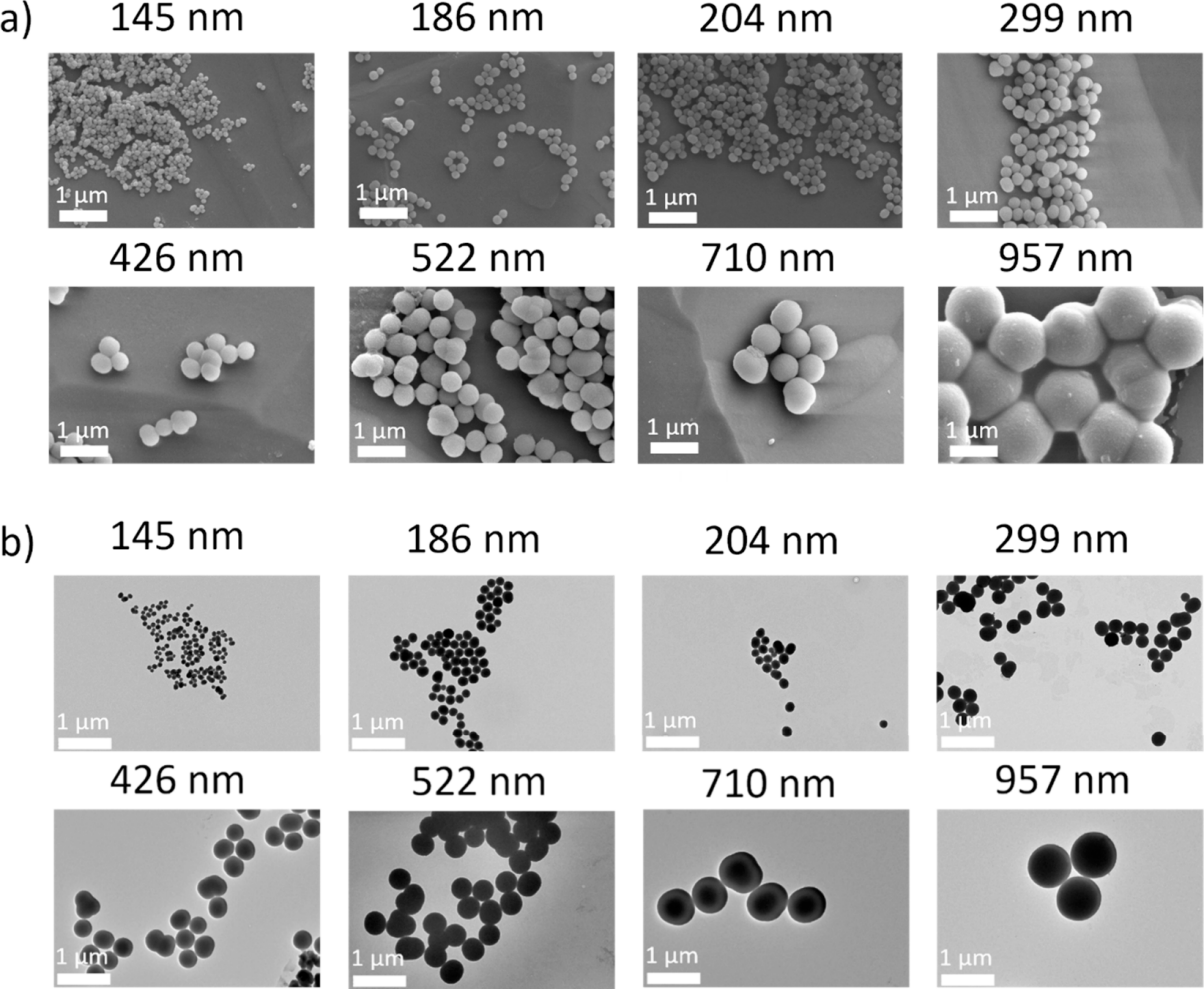
Morphological characterization of PDNPs: representative (a) SEM and (b) TEM images.

The AFM analysis was also exploited to determine how the nominal size of the nanostructures affected their average Young’s modulus. As shown in Figure 2a,b, five PDNPs of various nominal diameters were analyzed (145, 186, 204, 522, and 957 nm), and their average Young’s moduli were equal, respectively, to 2.3 ± 0.6, 2.3 ± 0.6, 3.2 ± 0.9, 4.1 ± 0.1, and 5.6 ± 0.2 MPa.

**Figure 2:**
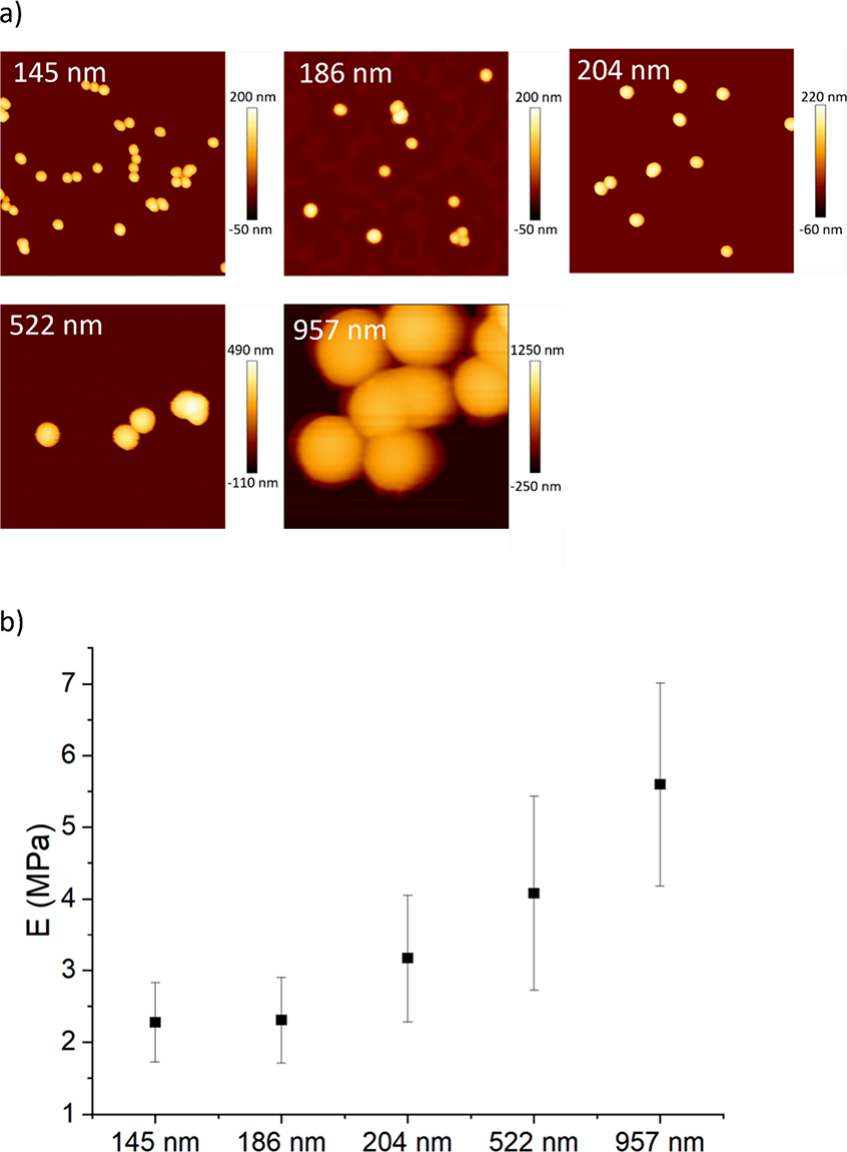
Characterization of PDNP Young’s modulus. (a) Representative AFM images of PDNPs of different nominal diameters. (b) Results of the Young’s modulus measurements.

Our analysis shows that bigger nanostructures have a higher Young’s modulus than smaller ones. Young’s modulus represents the elastic stiffness of a material, describing its ability to resist deformation under applied mechanical stress; in the case of nanoparticles, this parameter reflects the rigidity or flexibility of their internal structure, which can be influenced by factors such as composition, degree of polymerization, and hydration state.^52,53^ It is well known that the size of nanomaterials can affect their elasticity; however, the material constituting the nanostructures highly affects the dependence between nanomaterial size and their Young’s modulus. For example, it has been observed that, in the case of gold nanoparticles, smaller nanostructures have a higher Young’s modulus than bigger ones.^54^ A similar trend was observed for ZnO nanowires, where nanostructures with a smaller diameter showed higher values of Young’s modulus, while an opposite trend was observed for semiconductor nanomaterials.^55,56^

For photoacoustic applications, Young’s modulus is particularly relevant because the PA signal arises from rapid thermoelastic expansion following pulsed light absorption; therefore, measuring Young’s modulus provides useful insight into the mechanical contribution of PDNPs to their overall photoacoustic performance. Indeed, it is known that PA signal is highly affected by the elasticity of the imaged moieties, so that PAI is even exploited as a method for measuring the elasticity of materials, using a technique called quantitative photoacoustic elastography.^57,58^ Based on this, we hypothesize that differences in the elasticity of PDNPs of varying sizes are a key factor affecting their performance as PA contrast agents.

Stability studies were then performed by measuring the hydrodynamic diameter and PDI of the various PDNPs after incubation with full medium for 1, 24, and 48 h (Tables S1 and S2). We observed an increase in the average hydrodynamic diameters of the particles after incubation in full medium, which could be due to both the formation of a protein corona around the nanostructures and aggregation. Moreover, it was observed that smaller nanostructures maintained a rather low PDI (below 0.2 for particles up to 299 nm at all measured time points), while larger nanoparticles showed a higher PDI after incubation in full medium (up to 0.62 for PDNPs of 957 nm).

This size-dependent decrease in colloidal stability suggests that larger PDNPs are more prone to aggregation in biologically relevant environments, likely due to reduced Brownian stability and increased sedimentation tendencies. Such behavior may influence cellular uptake, biodistribution, and effective local concentration *in vitro* and *in vivo*, ultimately affecting their photoacoustic performance in complex biological systems. These findings therefore highlight that nanoparticle size impacts not only intrinsic optical and mechanical properties but also colloidal stability under physiological conditions, a critical parameter for translational applications.

The UV–vis–NIR absorption spectra of PDNPs with different nominal diameters (100 μg/mL in water) show a characteristic broadband optical absorption across the 680–970 nm range (Figure S3). All formulations exhibit a monotonic decrease in absorbance with increasing wavelength, consistent with the melanin-like optical behavior of polydopamine.^26,59,60^ Importantly, a clear size-dependent trend is observed, with larger nanoparticles displaying higher absorbance intensities throughout the entire spectral window compared to smaller ones. This indicates that increasing PDNP diameter enhances overall light absorption without altering the spectral profile shape. The preservation of broadband absorption combined with increased intensity for larger particles supports the hypothesis that size-dependent differences in photoacoustic performance are primarily related to variations in absorption magnitude rather than shifts in optical resonance. These results further corroborate the suitability of PDNPs as broadband contrast agents for near-infrared photoacoustic imaging applications.

## 4 Biocompatibility Studies

The biocompatibility of PDNPs was evaluated both *in vitro* on U87 cells and *in vivo* using zebrafish embryos to ensure that the concentrations selected for photoacoustic imaging experiments were within a biologically tolerated range. Live/dead assays performed on U87 cells exposed to PDNPs of eight nominal diameters (145–957 nm) at 100 and 200 μg/mL for 24 and 72 h demonstrated high cell viability across all experimental conditions (Figures S4–S6). No significant size-dependent or concentration-dependent cytotoxic effects were observed, even after prolonged exposure. Representative epifluorescence images confirmed the predominance of calcein-positive (live) cells and only a minimal presence of ethidium homodimer-1-positive (dead) nuclei under all tested conditions. Importantly, 200 μg/mL corresponds to the highest concentration used in the *in vitro* PAI experiments, and its lack of cytotoxicity supports the suitability of PDNPs for imaging applications in cellular systems.

*In vivo* safety was assessed by injecting zebrafish embryos with PDNPs of three representative sizes (145, 522, and 957 nm) at concentrations ranging from 50 to 300 μg/mL. At 50 μg/mL, the dose employed for *in vivo* PAI, embryo survival at 72 hpf was comparable to noninjected controls (Figure S7a). Although higher concentrations induced a progressive reduction in survival, this effect was concentration-dependent rather than clearly size-dependent. Morphological analysis at 120 hpf revealed no gross developmental abnormalities, and quantitative measurements of eye size and body length showed no significant differences compared to controls (Figure S7b–d). Furthermore, behavioral assessment demonstrated no alterations in swimming distance or velocity in treated larvae relative to untreated embryos (Figure S7e,f). Collectively, these results demonstrate that PDNPs exhibit a robust biocompatibility profile across a broad size range at the concentrations used for photoacoustic imaging, supporting their further development as label-free PAI contrast agents.

## 5 PAI in Different Configurations

As shown in Figure 3a, the PA signal generated by PDNPs of different nominal sizes was measured by exploiting hollow tubes submerged in water and filled with PDNP dispersions. All the tested particles showed a good PA signal compared to the background at all the tested wavelengths (680 to 970 nm). Representative PAI images of an aqueous solution not containing PDNPs and a dispersion of PDNPs of 957 nm in nominal diameter are shown in Figure 3b. The values of the PA signal originated from the PDNPs of increasing nominal sizes irradiated at 680 nm and at multiple wavelengths are instead shown in Figure 3c and Figure 3d, respectively. All the analyzed PDNPs had a maximum intensity of the PA signal generated around 680 nm, which decreased with the increase of the wavelengths of the incident laser. It is worth noting that the 957 nm PDNPs exhibit a localized increase in PA amplitude around ∼810 nm. This feature is not associated with a specific absorption peak of polydopamine, as confirmed by the corresponding UV–vis–NIR spectra, which show a monotonic broadband profile without discrete resonances in this region. We therefore attribute this isolated deviation to wavelength-dependent instrumental variability (e.g., minor fluctuations in laser pulse energy normalization or detector response) that become more evident for the highest-signal formulation. Importantly, this local variation does not affect the overall size-dependent trend observed across the spectral range.

**Figure 3:**
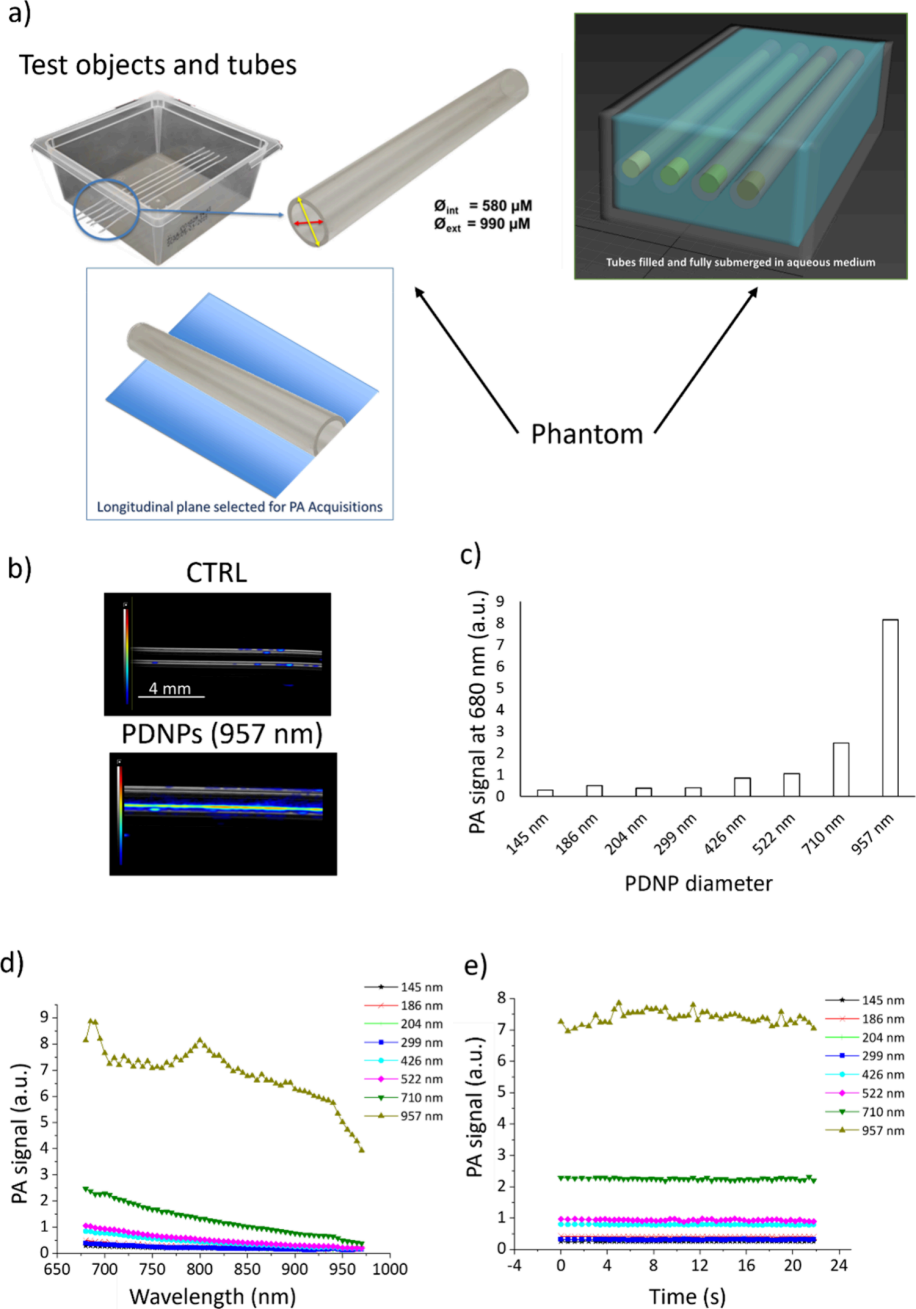
Analysis of PDNPs as PAI contrast agents in water dispersions. (a) A scheme depicting the setup for the PAI measurements. (b) Representative PAI images from photoacoustic acquisitions on test objects: The top image shows the PA signal generation from a water-filled tube, while the bottom image from a reference tube filled with PDNPs; grayscale represents the ultrasound signal, while the rainbow scale indicates the PA. (c) PA signal derived from PDNPs of different nominal diameters at 680 nm. (d) PA signal derived from PDNPs of different nominal diameters at various laser wavelengths. (e) Photostability of PDNPs during PAI at a fixed laser wavelength (705 nm).

The data shows how smaller nanostructures produce a lower PA signal than bigger ones. In particular, the measured PA signal at 680 nm ranged from 0.29 to 0.49 (all the data are expressed in arbitrary units) for particles of 145, 186, 204, and 299 nm, and it increased to 0.84 for PDNPs of 426 nm, to 1.15 for PDNPs of 522 nm, to 2.46 for PDNPs of 710 nm, and to 8.14 for PDNPs of 957 nm.

The photostability of the various nanostructures was then tested during PAI, exploiting a fixed laser irradiation at 705 nm as shown in Figure 3e. Over the analyzed time frame of 21.8 s, all the measured nanostructures maintained their photostability with variations in the generated PA below 3%. The only exception has been represented by PDNPs of 957 nm, which were slightly less photostable, presenting variations in the PA signal generated over the irradiation period up to approximately 12%.

We tested ICG as a positive control under identical acquisition conditions at 705 nm to provide a performance benchmark (PAI acquisition of ICG is shown in Figure S8). ICG exhibited the highest absolute PA signal (10.34 au) among all tested samples. However, several PDNP formulations, particularly the largest particles (957 nm), demonstrated better performance in terms of contrast (150.34 au vs 62.63 au for ICG) and signal stability, with %CV values in the same range (2.66% for 957 nm PDNPs vs 3.27% for ICG). While ICG showed higher raw PA intensity, larger PDNPs provided superior CNR and maintained competitive SNR values, indicating efficient and stable signal generation. Notably, PDNPs offer broadband absorption and improved photostability compared to organic dyes, suggesting that, despite slightly lower peak intensity than ICG, they represent robust and versatile alternatives for photoacoustic imaging applications. The values of PA signal, contrast, SNR, CNR, and %CV of the analyzed PDNPs and ICG are reported in Table S3.

The ability of PDNPs to act as contrast agents for PAI was tested in an *ex vivo* model consisting of chicken breasts (Figure 4a) injected with dispersions of PDNPs of different nominal sizes First, we tested the effect of PDNP concentration on the obtained PA signal by injecting 204 nm PDNPs. As shown in Figure 4b, the concentration of PDNPs plays a pivotal role in determining the obtained PA signal, with the PA SNR and CNR decreasing with the increase in the dilution factor of the PDNP dispersions. Figure 4c shows some representative PA images depicting PDNPs of different sizes injected into the chicken tissue, demonstrating that PDNPs of all the tested nominal diameters can be easily detected through PAI. The graph in Figure 4d shows the PA signals obtained from PDNPs of different nominal diameters at various wavelengths of laser irradiation (from 680 to 970 nm). Once again, the PA signal obtained from the nanostructures depended on their nominal sizes, with smaller nanostructures performing worse as PAI contrast agents than larger ones, with the exception of 957 nm PDNPs, which performed worse than the smaller nanostructures. Figure 4e shows the photostability of PDNPs injected in chicken breast and imaged through PAI for 11.3 s (irradiation wavelength 705 nm). All the analyzed nanostructures showed relatively good photostability, with variations in the obtained PA signal below 8% over all the irradiation time frame. A control tissue sample injected only with PBS showed no appreciable PAI signal relative to the surrounding tissue (Figure S9).

**Figure 4:**
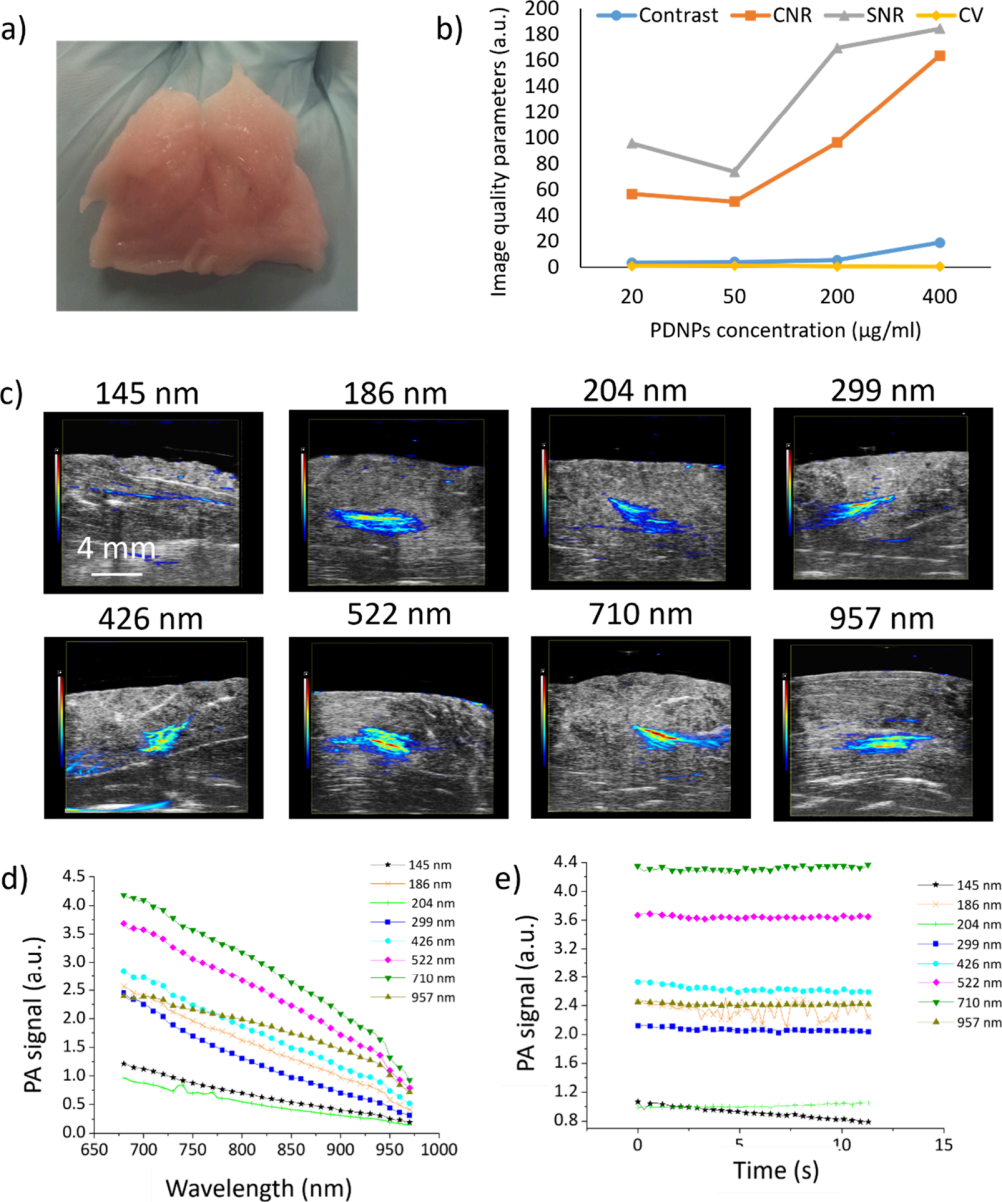
Analysis of PDNPs as PAI contrast agents in an *ex vivo* model (chicken breast). (a) Representative picture of the tissue exploited as *ex vivo* model (a chicken breast fragment). (b) Contrast, CNR, SNR, and CV derived for PDNPs of 200 nm injected into the samples. (c) Representative PA images obtained with PDNPs of different nominal sizes injected into chicken tissue. (d) PA signal derived from PDNPs of different nominal diameters at various laser wavelengths. (e) Photostability of PDNPs during PAI at a fixed laser wavelength (705 nm).

PDNPs as contrast agents in PAI were tested on U87 cells spheroids (Figure 5a). Figure 5b shows PA representative images of U87 spheroids treated with and without PDNPs of various nominal diameters. Figure 5c shows the PA signals obtained after irradiation with different laser wavelengths (from 680 to 970 nm). All the spheroids treated with nanostructures showed a PA signal higher than the nontreated control spheroids. In these tests, the obtained PA signal was not wholly dependent on the PDNP diameters: The smallest nanostructures (186 nm) were indeed the worst-performing class in terms of generated PA signal (maximum value 3.30 a.u); conversely, the best-performing nanoparticles were PDNPs of 299 and 426 nm (maximum PA signal of 96.79 and 82.87 au, respectively), which outperformed larger nanostructures such as PDNPs of 710 and 957 nm (maximum PA signal of 38.24 and 45.03 au, respectively). In Figure 5d, the photostability analysis of the PDNPs administered to the spheroids imaged through PAI for 8.7 s is reported (irradiation wavelengths equal to 705 nm). Once again, all the analyzed PDNPs showed good photostability with changes in the generated PA signal below 7% for all the analyzed experimental conditions.

**Figure 5:**
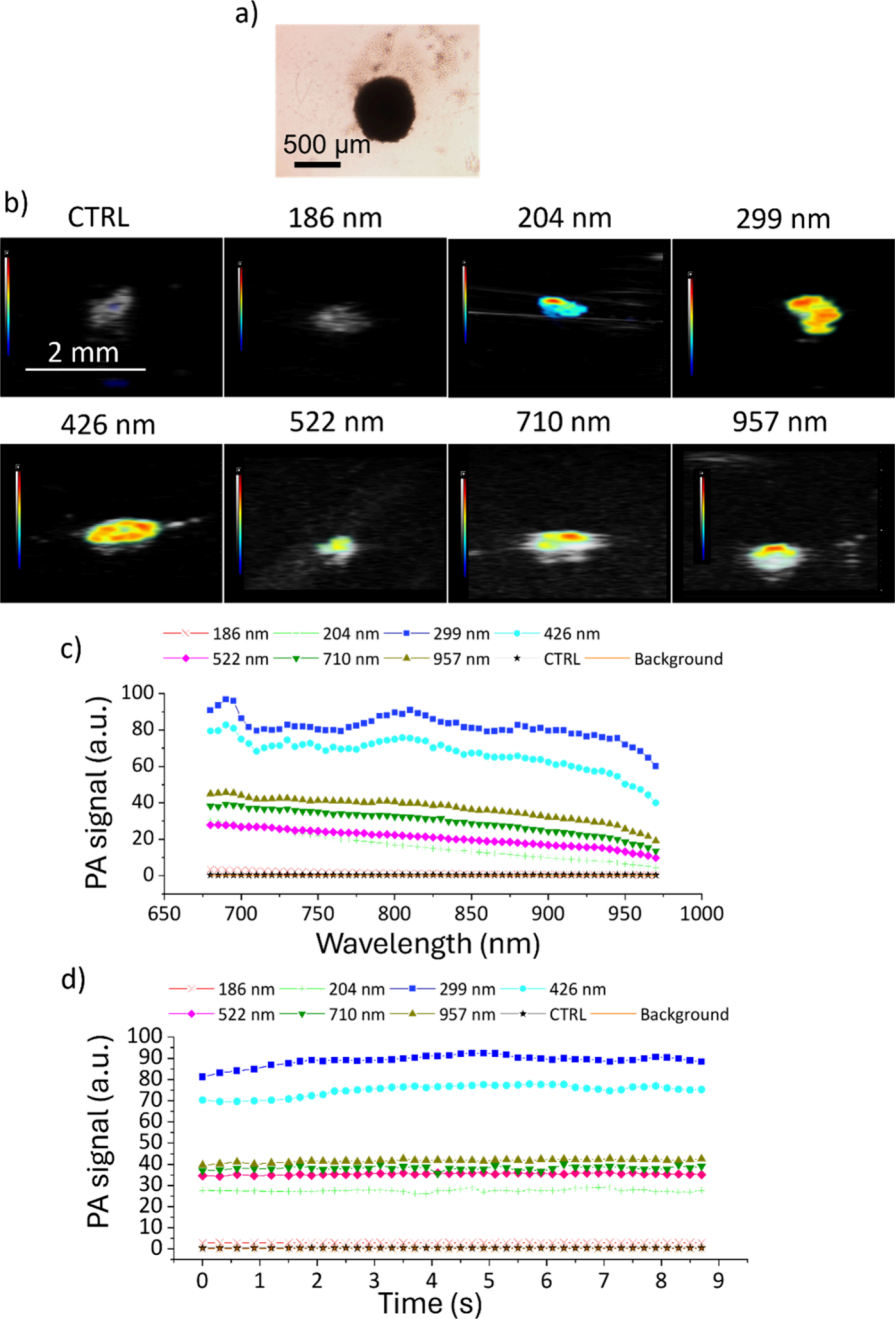
Analysis of PDNPs as PAI contrast agents in a cellular model (spheroids of U87 cells). (a) Representative bright-field image of a U87 spheroid. (b) Representative PA images obtained with PDNPs of different nominal sizes administered to U87 spheroids. (c) PA signal derived from PDNPs of different nominal diameters at various laser wavelengths. (d) Photostability of PDNPs during PAI at a fixed laser wavelength (705 nm).

The PA properties of PDNPs were further tested on an *ex ovo* model consisting of fertilized quail eggs (schema in Figure 6a) injected with PDNPs of three different nominal diameters (145, 522, and 957 nm, respectively). Figure 6b shows a picture of the vascularized quail embryo (i), a 3D reconstruction of the embryos obtained through PAI (ii), and a 3D reconstruction of the embryo and of the surrounding blood vessels obtained through PAI (in gray the embryo tissues, in red oxygenated hemoglobin). Figure 6c shows the PA signal obtained from the PDNPs of three different nominal diameters irradiated with a laser at different wavelengths. In the context of the *ex ovo* tests, it was confirmed that bigger nanostructures performed better as PA contrast agents than smaller ones. In particular, in the NIR window (750–900 nm) PDNPs of 957 nm generated a PA signal 50% higher than PDNPs of 522 nm, and almost 100% higher than PDNPs of 145 nm (Figure 6d).

**Figure 6:**
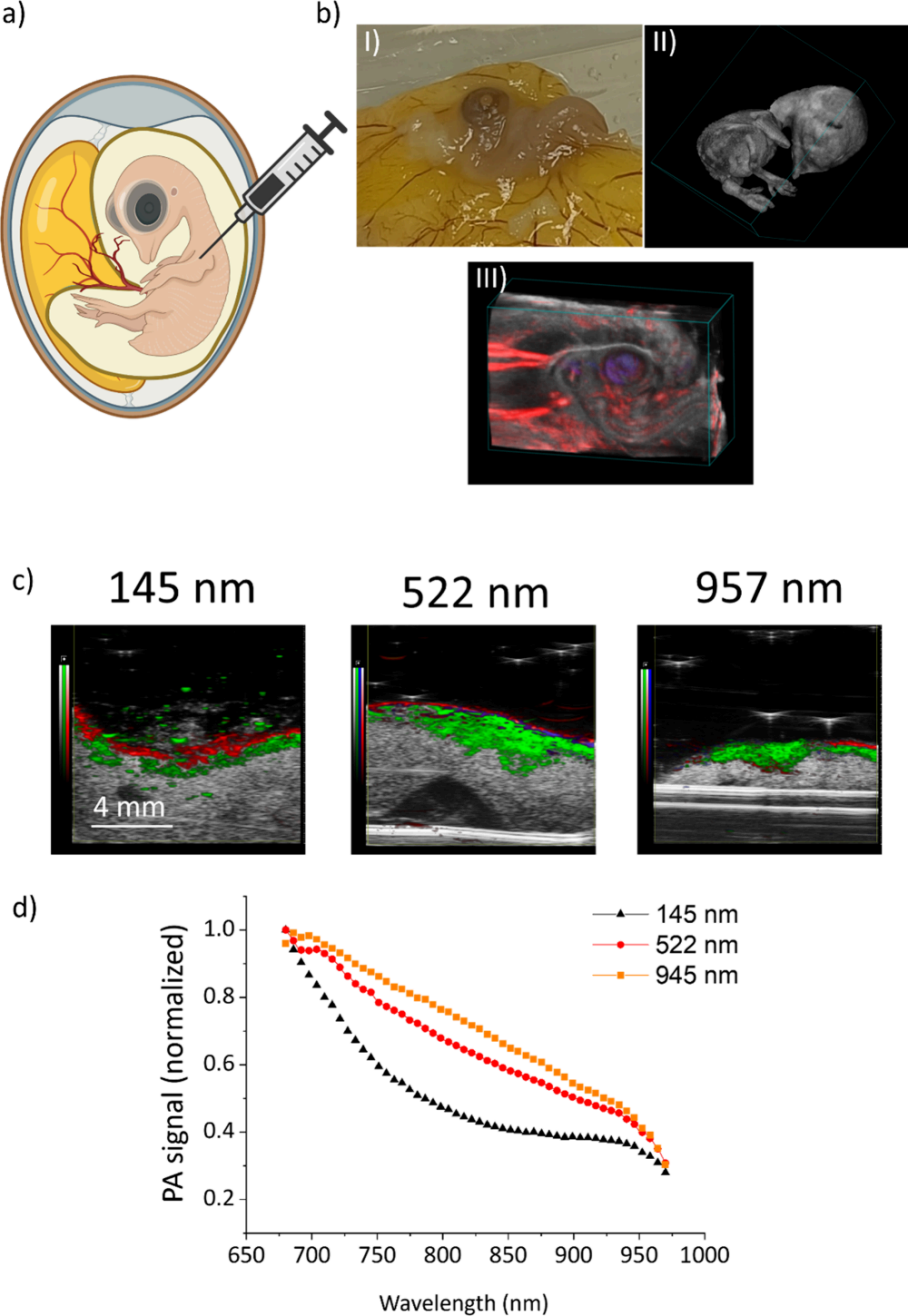
Analysis of PDNPs as PAI contrast agents in an *ex ovo* model (fertilized quail eggs). (a) Schema depicting the *ex ovo* model. (b) Representative picture (I), representative PA image (II), and representative 3D reconstruction (III) of a quail embryo (in gray the embryo tissue, in red oxygenated hemoglobin). (c) Representative images obtained with PDNPs of different nominal sizes injected in the eggs (in gray embryo tissues, in red oxygenated hemoglobin, in green PDNPs). (d) Normalized PA signal derived from PDNPs of different nominal diameters at various laser wavelengths.

The PA signal originating from PDNPs was eventually tested on an *in vivo* model represented by zebrafish embryos. The analyzed animals were injected into the yolk sac as depicted in Figure 7a and then fixed with PFA and imaged through PAI. All the tested nanostructures (145, 522, and 957 nm) were able to generate a PA signal in the animals higher than that one of control nontreated animals (representative PA images in Figure 7b). In particular, PDNPs of 957 nm in nominal diameter generated a maximum PA signal of 1.81 au, PDNPs of 145 nm a signal of 1.94 au, and PDNPs of 522 nm a signal of of 3.51 au. In the context of the *in vivo* experiments, however, the smallest and the biggest nanostructures (145 and 957 nm, respectively) generated a similar PA signal, while PDNPs with a medium nominal size (522 nm) performed as the best PAI contrast agents (Figure 7c). Also concerning *in vivo* experiments, all the tested nanostructures presented good photostability, with variations in the generated PA signal below 12% (Figure 7d).

**Figure 7:**
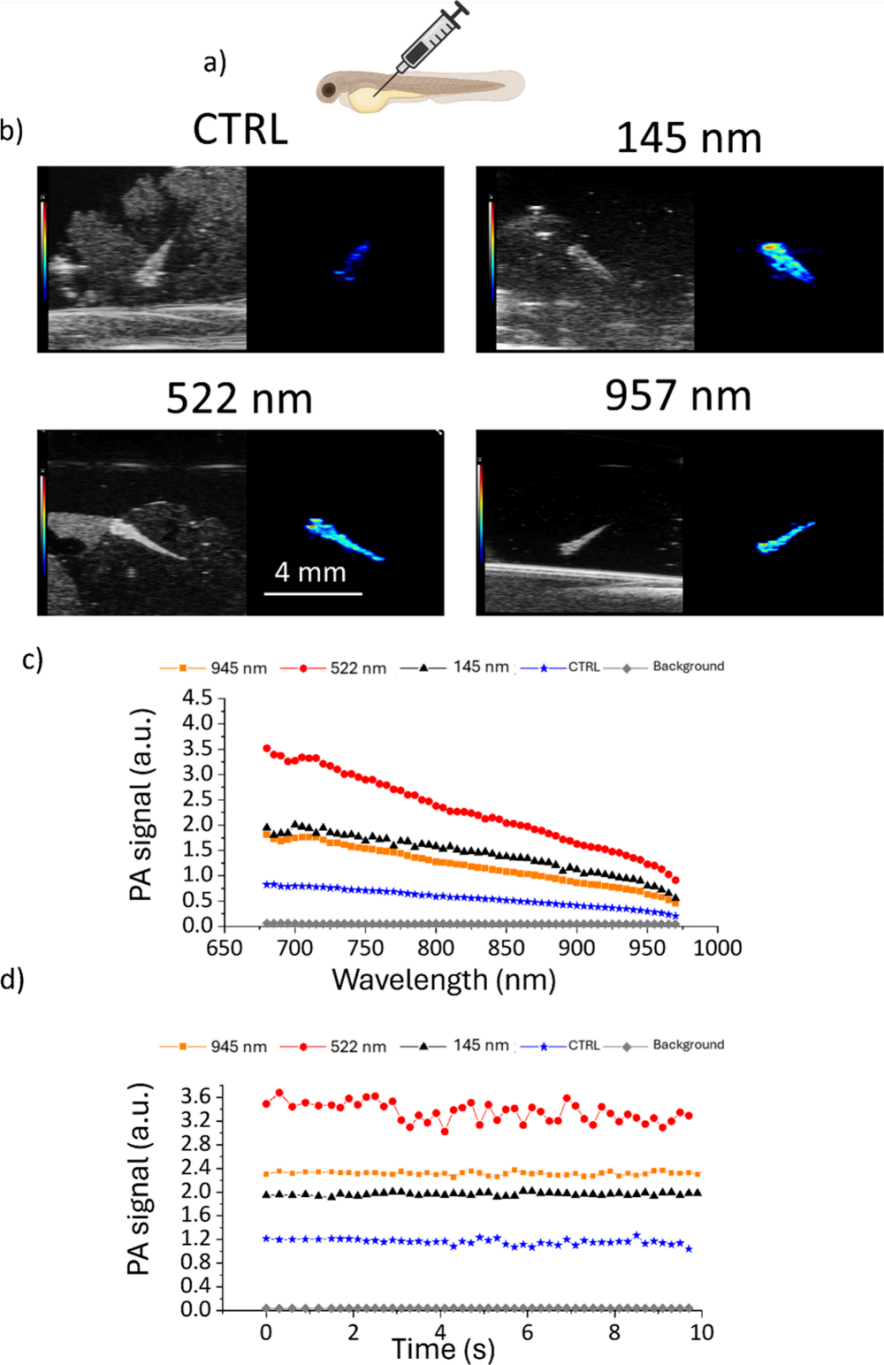
Analysis of PDNPs as PAI contrast agents in an *in vivo* model (zebrafish embryos). (a) Scheme depicting the injection of PDNPs in the yolk of zebrafish embryos. (b) Representative PAI images of zebrafish injected with PDNPs of different nominal diameters. (c) PA signal derived from PDNPs of different nominal diameters at various laser wavelengths. (d) Photostability of PDNPs during PAI at a fixed laser wavelength (705 nm).

Data collected exploiting nanostructures in water dispersions demonstrates a dependence between nanoparticle sizes and PA signals generated, with bigger nanostructures performing as better contrast agents. We hypothesize that this effect is due to two key factors: the differences in the elasticity and in the light absorption properties of PDNPs of different sizes. We demonstrated in previous work that the light absorption of PDNPs depends on their size, with bigger nanoparticles absorbing at higher extent in the NIR region with respect to the smaller ones.^5^ Moreover, we also demonstrated that the photothermal conversion properties of PDNPs depend on their sizes, with bigger nanostructures reaching higher temperatures than smaller ones when irradiated with the same laser source.^5^ Then, it is expected that bigger PDNPs, being able to heat up more than smaller ones under irradiation, can undergo a higher degree of thermal expansion, thus generating a higher PA signal. These experimental results align with our computational model, where a direct dependence between PDNP size and generated PA signal was confirmed.

It has been observed that the nanostructure size can affect the PA signal obtained during PAI, exploiting other classes of nanomaterials such as gold nanorods and gold nanoparticles.^61,62^ However, it is worth mentioning that the physical mechanism behind the ability of PDNPs and noble metal nanostructures to act as PA contrast agents is entirely different, thus making it hard to compare the two classes of nanomaterials. Noble metal nanostructures such as gold nanorods can be exploited for PAI thanks to their plasmon resonance properties, and changes in their shape and size can affect both their photothermal and PA properties. However, gold nanorods and other plasmon resonant nanomaterials have a limited wavelength window of efficiency, corresponding to their plasmon resonance peak.^63^ Changes in the size and shape of plasmon resonant nanomaterials cause a shift in their plasmon resonant peak, thus affecting the light wavelength at which they can act as photothermal converters and PA contrast agents.^61,62^ This work demonstrates that PDNPs can absorb light and act as PA contrast agents in a broader range of wavelengths. The changes in PDNP diameters seem to affect the absorption coefficient of the nanostructures and, in turn, the generated PA signal, but not the absorption wavelength. Unlike gold nanorods, whose photoacoustic performance relies on localized surface plasmon resonance (LSPR) with a narrow and shape-dependent absorption peak, PDNPs exhibit broadband, structure-independent optical absorption arising from their melanin-like electronic structure.^61–63^ While gold nanorods require precise control of the aspect ratio to tune the resonance wavelength into the near-infrared region, PDNPs inherently absorb across a wide spectral range (approximately 600–1000 nm), allowing flexible excitation without geometric reshaping.^61–63^ Moreover, PA efficiency in gold nanorods is strongly influenced by plasmon damping, surface chemistry, and reshaping under laser irradiation, whereas PDNPs demonstrate high photostability and do not rely on resonant plasmonic oscillations. From a size-effect perspective, gold nanorods typically optimize PA response through aspect ratio modulation rather than simple size scaling, while PDNPs show a direct correlation between diameter and intrinsic photothermal conversion under homogeneous conditions.^61–63^ In terms of biocompatibility, PDNPs are organic, metal-free, and biodegradable in oxidative environments, whereas gold nanorods may require surface functionalization to mitigate long-term persistence and potential cytotoxicity. Collectively, these differences highlight PDNPs as broadband, structurally robust, and intrinsically biocompatible alternatives to plasmonic nanomaterials for photoacoustic imaging applications.

It is important to note that the optical absorption properties and photothermal conversion efficiency of PDNPs have already been systematically investigated in a previous study of our group.^5^ In that work, we demonstrated that the broadband absorption profile of PDNPs in the visible–NIR range is largely independent of size, while the photothermal conversion efficiency exhibits a moderate size-dependent increase. These findings provide a mechanistic background for the present results, supporting the conclusion that the stronger photoacoustic responses observed for larger PDNPs are not primarily due to changes in absorption but instead arise from the combined effects of photothermal efficiency and mechanical factors such as elasticity.

Similar results were obtained by exploiting an *ex vivo* model based on chicken breasts, where, once again, bigger nanostructures generated the highest PA signal. However, as previously mentioned, the largest tested nanostructures (957 nm) did not perform as the best PA contrast agents when injected in the tissues. This could be due to the relatively low colloidal stability of PDNPs at this nominal size, as described in a previous publication from our group.^5^ PDNP PA properties were then tested on U87 spheroids, where, as already commented, there was no direct relation between nanostructure nominal size and PA signal. We hypothesized that this phenomenon could be due to the different degrees of internalization of PDNPs of different nominal diameters. In previous work from our group, we already described how smaller PDNPs are more easily internalized by glioblastoma cells;^5,3^ therefore, the PA signal derived from PDNPs administered to spheroids is probably depending also on the relative uptake by the cells. Concerning the *ex ovo* model, it was confirmed that the trend is for bigger nanostructures to produce higher PA signal than smaller ones.

The CAM model based on fertilized quail embryos has gained attention as a low-cost, high-throughput platform to study angiogenesis during early development and a system for developing xenografts of various forms of human cancer.^64^ CAM models have also been exploited to study nanostructure properties such as biocompatibility, targeting, angiogenic activity, drug delivery and release, tumor accumulation, and anticancer efficiency.^65^ In the context of PAI, chicken embryos have been exploited to prove the technique ability to image the developing embryos and of the surrounding blood vessels in a label-free manner.^66^ However, to the best of our knowledge, the CAM model has never been exploited to test the PA properties of nanostructures. Our work demonstrates that PDNPs can be easily localized inside the CAM model and its potential to allow the assessment of the ability of nanostructures to act as PAI contrast agents within a vascularized biological environment.

Lastly, in the *in vivo* model (zebrafish embryos), it was observed that the PDNP diameters were not predictive of the obtained PA signal, with the biggest nanostructures (957 nm) performing as the worst contrast agents. Zebrafish, having a similar early development and sharing more than 87% of the genome with humans, have gained much attention as an alternative *in vivo* model in comparative biology and disease research.^67^ Zebrafish embryos are also increasingly being investigated as a model to test nanomaterial biocompatibility and effects.^67,68^ In the context of PAI, zebrafish embryos have been exploited to demonstrate the potential of the technique to acquire high-resolution label-free 3D images of developing embryos relying on the intrinsic optical absorption of the animal tissues;^69,70^ however, the application of PA contrast agents such as dyes and nanostructures could greatly improve the PAI of zebrafish embryos, broadening the applicability of this technique for development and nanotoxicological studies. Our data indicate that, independently from their size, PDNPs can always generate a PA signal higher than the endogenous chromophore present in the embryos with a relatively good photostability. These results suggest that the PDNP size is not the only parameter affecting the obtained PA signal; other factors, such as colloidal stability and biodistribution within blood vessels and tissues, play also probably a relevant role in the generated PA signal.

The different trends observed between aqueous/*ex vivo* models and *in vivo* experiments can be rationalized by considering the role of biological interactions. In living systems, factors such as cellular uptake, nanoparticle aggregation in complex fluids, and biodistribution patterns strongly affect the effective PA signal. Smaller PDNPs are more prone to homogeneous internalization, whereas larger ones may remain extracellular or cluster in specific compartments, resulting in signal heterogeneity.^5^ Furthermore, the formation of a protein corona, recently characterized in detail for PDNPs, can alter their colloidal stability and optical response *in vivo*. Together with our previous findings showing PDNP biocompatibility, BBB crossing, and size-dependent cellular interactions,^5,71^ these considerations suggest that the *in vivo* PA behavior of PDNPs reflects not only intrinsic size-dependent properties but also their dynamic biological fate. In this context, it is also important to note that the largest PDNPs (957 nm) underperformed in *ex vivo* and *in vivo* models compared to their strong PA response in aqueous suspension. This effect can be explained by considering the impact of colloidal stability, tissue penetrability, and biodistribution. Very large nanoparticles are more prone to aggregation and sedimentation in complex biological fluids, which reduces their effective dispersion. In addition, their size limits tissue penetration and homogeneous distribution, restricting access to certain anatomical compartments. These limitations, combined with the influence of protein corona formation at large diameters, are likely to reduce the bioavailability of the largest particles *in vivo*.^5,71^ Therefore, while larger PDNPs maximize intrinsic photoacoustic efficiency in homogeneous environments, their translation into biological systems is constrained by stability and transport factors, further underscoring the role of biological interactions in determining *in vivo* PA performance.

## 6 Computational Model of PDNPs as PAI Contrast Agents

To model the photoacoustic pressure values produced by PDNPs in both the far and near fields, and evaluate the differences among nanoparticles of different sizes, we generated two finite element models using the COMSOL Multiphysics software. These finite element models were based on the previously published works of Handte et al. and Davletshin et al.^47,49^

The first far-field “macroscopic” model simulated PDNP dispersions as fluids with different absorption coefficients related to their diameter (Figure S10. Values of size-related absorption coefficients for PDNP suspensions were taken from previously published work from our group about the photothermal conversion properties of PDNPs of different diameters.^5^ The computational macroscopic simulation was exploited to calculate quantitative data concerning the measured PA signals detected in an experimental setting with aqueous dispersions of PDNPs. As shown in Figure S10a, the geometrical configuration of the simulation was directly modeled basing on the experimental setup described in Section 2.7, where PVC tubes filled with PDNP dispersions were immersed in a water-filled plastic box. The pulsed NIR-laser source (wavelength 705 nm) was placed above the plastic container (not shown in the numerical model), forming a cylindrical spot around the tubes placed in the water below it, and with the axes of the laser spot matching the tube axes. This spot shape was chosen to guarantee a homogeneously distributed light source over the entire surface of the various samples. According to the experimental specifications, the employed laser had a peak energy of ≈50 mJ, with a pulse width τ*w* < 10 ns and a pulse rate of 20 Hz, which translates to a pulse duration of 50 ms. Assuming that the thermal relaxation time of PDNPs is much shorter than the pulse period, the PA process was modeled as thermally confined.^50^

Based on this, the numerical simulation was limited to a single laser pulse, thus reducing the computational cost of the simulation. Figure S10b shows the 3D map of the calculated PA signal (on the left) obtained by irradiating an aqueous dispersion of PDNPs of 957 nm in diameter. The modeled PA map, with pressure values normalized to the maximum, showed a similar trend to the experimental data (on the right), with the PA signal localized at the edges of the experimental setup, where most of the viscous loss occurs. Figure S10c represents experimental PA data obtained from the irradiation of PDNP dispersions of different nominal diameters over the time; as previously described, the pulse frequency allows for the thermal relaxation of the system between consecutive laser pulses, which results in an almost constant PA signal over the time for all the PDNP dispersions tested in this work. Figure S10d shows the simulated PA signal obtained from a single irradiation, changing the nominal sizes of the PDNPs and, thus, their absorption coefficient. The results obtained from experimental analysis and computational model show that the PA signal derived by the laser irradiation of PDNPs can be enhanced by increasing the size of the nanostructures.

The generation of PA waves in a nanostructure dispersion differs from that one in a pure light-absorbing liquid or solid due to the anisotropy of the system thermal and optical properties.^51^ Thus, based on previous literature on gold nanoparticle PA signal analysis, we implemented a second near-field “nanoscale” simulation.^48^ This computational model was chosen to calculate the PA response of a single PDNP in an aqueous dispersion. In particular, we wanted to assess the mechanical and thermal response of PDNPs when exposed to a single NIR-laser pulse to calculate the pressure gradient generated at the interface between the nanostructures and the surrounding environment due to their thermally induced mechanical expansion. Figure 8a shows the geometrical model created in the simulation environment, where a nanostructure, assumed to be an elastic solid, is placed in the center of a discrete volume of water, which is treated as a fluid. To calculate the acoustic wave generating from the nanoparticle and diffusing to the surrounding medium, the following constraints are considered: (1) the transient heat transfer and the temperature increase caused by the absorbed incident energy, where the temperature is exploited as a coupling parameter; (2) the structural mechanics for linear thermal expansion, stress, and strain calculation for the input as a boundary condition; (3) the acoustic-structure interaction for acoustic pressure wave propagation calculation. Figure 8b and Figure 8c illustrate the results of the temperature increments and of the associated thermal expansions caused by a single laser pulse of 10 ns; the initial temperature was set at 303 K. As expected, PDNPs with larger diameters are associated with a higher photothermal conversion ability than the smaller ones, undergoing an increment of 1 K in a few nanoseconds (Figure 8b). Temperature increases translate into a thermoelastic expansion of the nanoparticle surface in the order of picometers, with the maximum obtained for PDNPs of a nominal diameter of 957 nm, reaching 16 pm of surface displacement. The PA signal generated from the irradiation of the nanoparticles is calculated by imposing the PDNP surface displacement as a boundary parameter in the acoustic model. The 2D color pressure map originated from the PDNP surface displacement is represented in Figure 8d illustrating a progressive increase in the pressure from the solid–fluid interface to the fluid (pressure values are associated with increases or decreases compared to the equilibrium condition *p*_0_ = 1 MPa). Figure 8d shows the spatial distribution of the pressure around the nanostructure. At 5000 nm from the nanostructure center, the pressure increases by about 130 Pa compared to the equilibrium state (Figure 8e); increasing nanoparticle diameters causes the generation of enhanced PA signals. The pressure value at 5000 nm from the nanoparticle surface for different PDNP nominal diameters is plotted in Figure 8f, showing a similar trend as in the macroscopic model. From the numerical results obtained by exploiting the nanoscopic and macroscopic models, it is again evident that increasing PDNP diameters would lead to an enhanced PA signal. From the near-field simulation, it can be claimed that the diameter of the nanostructures affects the photothermal conversion efficiency of these nanomaterials and, thus, the thermal expansion properties of the PDNPs at the base of the PA signal generation.

**Figure 8:**
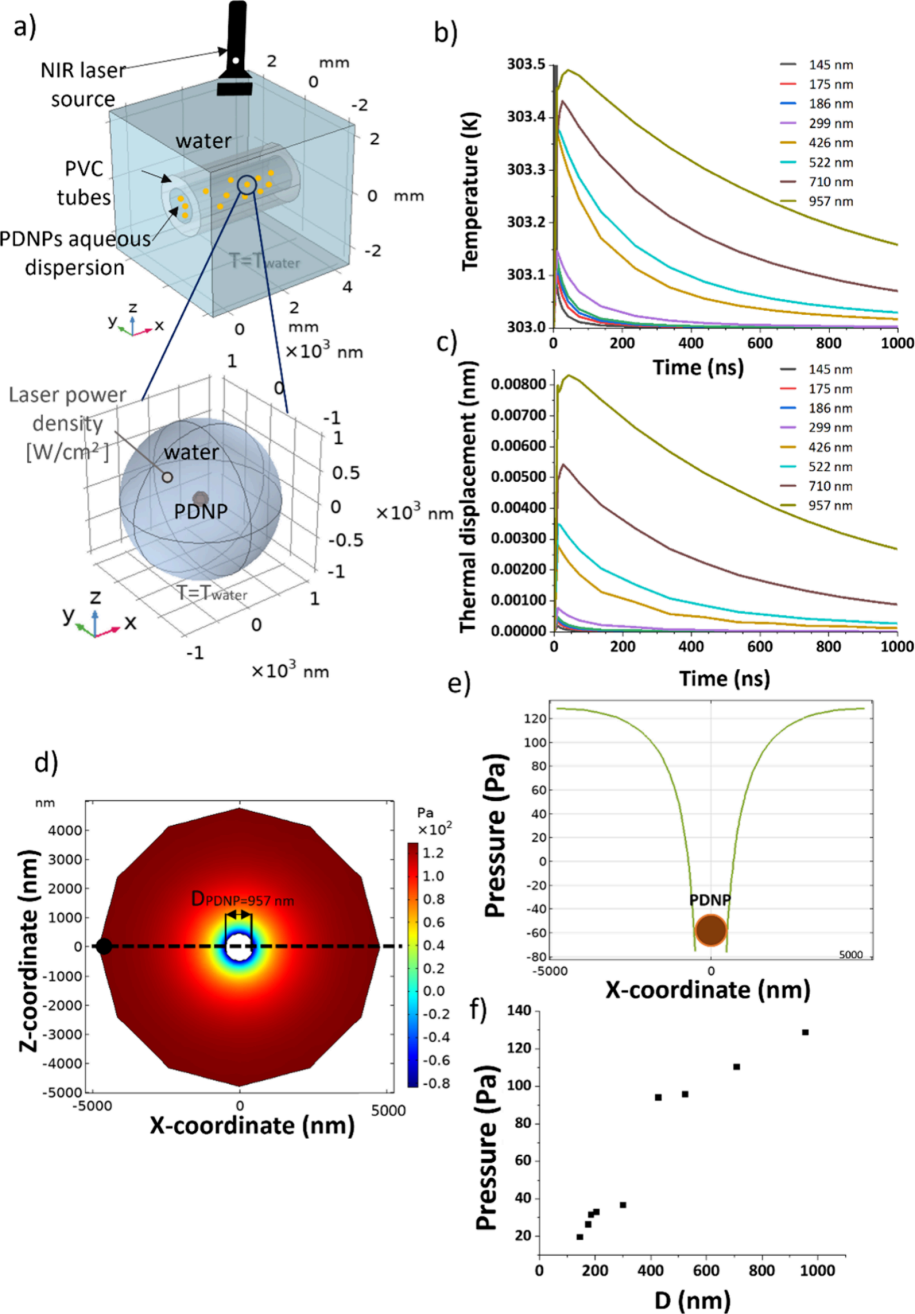
COMSOL model of PA phenomena upon PDNP laser irradiation. (a) Geometrical model in COMSOL. (b) Temperature increment after a single laser pulse (10 ns) for different PDNP nominal sizes and (c) consequent thermal expansions. (d) 2D map of the acoustic pressure from the PDNP surface to the surrounding water volume. (e) Profile of the pressure from the PDNP surface to the surrounding water volume (*z* = 0; *y* = 0). (f) Pressure value at *x* = 5000 nm, *y* = 0, *z* = 0 for PDNPs of different nominal sizes.

Importantly, it should be emphasized that both the far-field and near-field models were developed under homogeneous aqueous conditions and therefore describe the intrinsic photothermal and thermoelastic behavior of PDNPs in an idealized environment. The simulations do not account for biological complexity factors such as protein corona formation, nanoparticle aggregation, heterogeneous tissue optical scattering, size-dependent cellular uptake, biodistribution, or confinement within biological compartments. Consequently, while the model accurately predicts the size-dependent “larger particle–stronger signal” trend observed in aqueous dispersions, it is not intended to reproduce deviations occurring in *ex vivo* or *in vivo* systems. In particular, the reduced performance of 957 nm PDNPs in tissue and the superior behavior of intermediate-sized particles (e.g., 522 nm) in zebrafish embryos likely arise from size-dependent colloidal stability, aggregation dynamics, and biological distribution effects, which are beyond the current modeling framework. Incorporating such biological variables would require multiphysics simulations coupling optical scattering, transport phenomena, and nanoparticle–cell interactions, which fall outside the scope of the present study. Therefore, the computational model should be interpreted as a mechanistic tool describing the intrinsic photothermal-to-acoustic conversion efficiency of PDNPs as a function of size, rather than as a predictive model of full biological performance. The divergence observed in complex biological systems highlights the additional role of colloidal stability and biodistribution, as experimentally supported by our newly included stability data.

## 7 Conclusions

In this work, we presented the first complete characterization of the potential of PDNPs in PAI. Our results can be summarized in some important key take-home messages. (*i*) PDNPs from 100 to 1000 nm can produce a strong PA signal, making them good label-free contrast agents for PAI applications in biological environments. Moreover, all the nanostructures present good photostability, maintaining a constant PA signal during the PAI procedure. (*ii*) PDNP diameters are pivotal in determining their ability to act as PA contrast agents. In most cases, bigger nanostructures can generate a higher PA signal than smaller ones. (*iii*) PDNP size is not the only parameter affecting the intensity of the generated PA signal. When PDNPs are administered to complex biological environments, other parameters, like the colloidal stability of the nanoparticles and their biodistribution, can also play an important role in determining their efficiency as PAI contrast agents.

Based on the experimental results described in this work, a predictive computational simulation of the PA properties of PDNPs has been developed, that we are confident it can be exploited as a guideline for developing polydopamine-based PAI contrast agents. Future perspectives will involve testing how other parameters of PDNPs can affect their PA signal efficiency, such as shape and/or porosity. In addition, although zebrafish and avian embryo models demonstrate favorable short-term biocompatibility, comprehensive long-term biodistribution, metabolic fate, and clearance studies in mammalian models will be essential to fully assess the translational potential of PDNPs. Finally, other biological models, such rodents *in vivo* models, will be exploited to test the PA properties of PDNPs, envisioning future theragnostic applications.

## Supplementary Material



## Data Availability

All data are available from the authors upon reasonable request.

## Notes

DLS and ζ-pot analysis of PDNPs (Figures S1 and S2); UV–vis–NIR absorption spectra of PDNPs of different diameters (Figure S3); cytotoxicity evaluation on U87 cells including quantitative Live/Dead assay and representative fluorescence images (Figures S4–S6); zebrafish embryo toxicity and behavioral assessment (Figure S7); photoacoustic characterization of ICG as reference contrast agent (Figure S8); control photoacoustic experiments in chicken breast tissue (Figure S9); computational modeling of the photoacoustic response of PDNP dispersions (Figure S10); hydrodynamic diameter and PDI of PDNPs after incubation in cell culture medium (Tables S1 and S2); and quantitative photoacoustic parameters for PDNPs and ICG (Table S3) (PDF)

## #

Current address: Shenzhen University of Advanced Technology, Gongchang Road 1, Guangming District, 518107 Shenzhen, People’s Republic of China
