# Supplementary material for: Polydopamine Nanoparticles as Label-Free Contrast Agents in Photoacoustic Imaging In Vitro and In Vivo
Source: ACS Appl Opt Mater. 2026 Mar 23;4(4):1051–67. doi: 10.1021/acsaom.5c00637 (PMC13131747; doi:10.1021/acsaom.5c00637)
Supplement: Supplementary file 1 [file ot5c00637_si_001.pdf]

Supporting information for

Polydopamine nanoparticles as label-free contrast agents in photoacoustic imaging *in vitro* and *in vivo*

Matteo Battaglini[1,*], Paolo Armanetti[2], Alessio Carmignani[1], Claudia Catarinicchia[2], Valentina Naef[3,4], Margherita Montorsi[5,#], Marie Celine Lefevre[1], Claudio Canale[6], Davide Odino[6], Luca Menichetti[2], Filippo Maria Santorelli[3,4], Gianni Ciofani[1,7,*]

[1] Istituto Italiano di Tecnologia, Smart Bio-Interfaces, Viale Rinaldo Piaggio 34, 56025 Pontedera, Italy

[2] National Research Council, Institute of Clinical Physiology, Via Giuseppe Moruzzi 1, 56124 Pisa, Italy

[3] IRCCS Stella Maris Foundation, Unit of Neurobiology, Via dei Giacinti 2, 56128 Calambrone (Pisa), Italy

[4] IRCCS Stella Maris Foundation, Molecular Medicine and Neurogenetics, Via dei Giacinti 2, 56128 Calambrone (Pisa), Italy

[5] National Research Council, Institute for the Chemical and Physical Processes, Largo Pontecorvo 3, 56127 Pisa, Italy

[6] University of Genova, Department of Physics, Via Dodecaneso 33, 16146 Genova, Italy

[7] Scuola Superiore Sant'Anna, Health Sciences Interdisciplinary Center, Piazza Martiri della Libertà 33, 56127 Pisa, Italy

[#] Current address: Shenzhen University of Advanced Technology, Gongchang Road 1, Guangming District, 518107 Shenzhen, People's Republic of China

*matteo.battaglini@iit.it; gianni.ciofani@iit.it

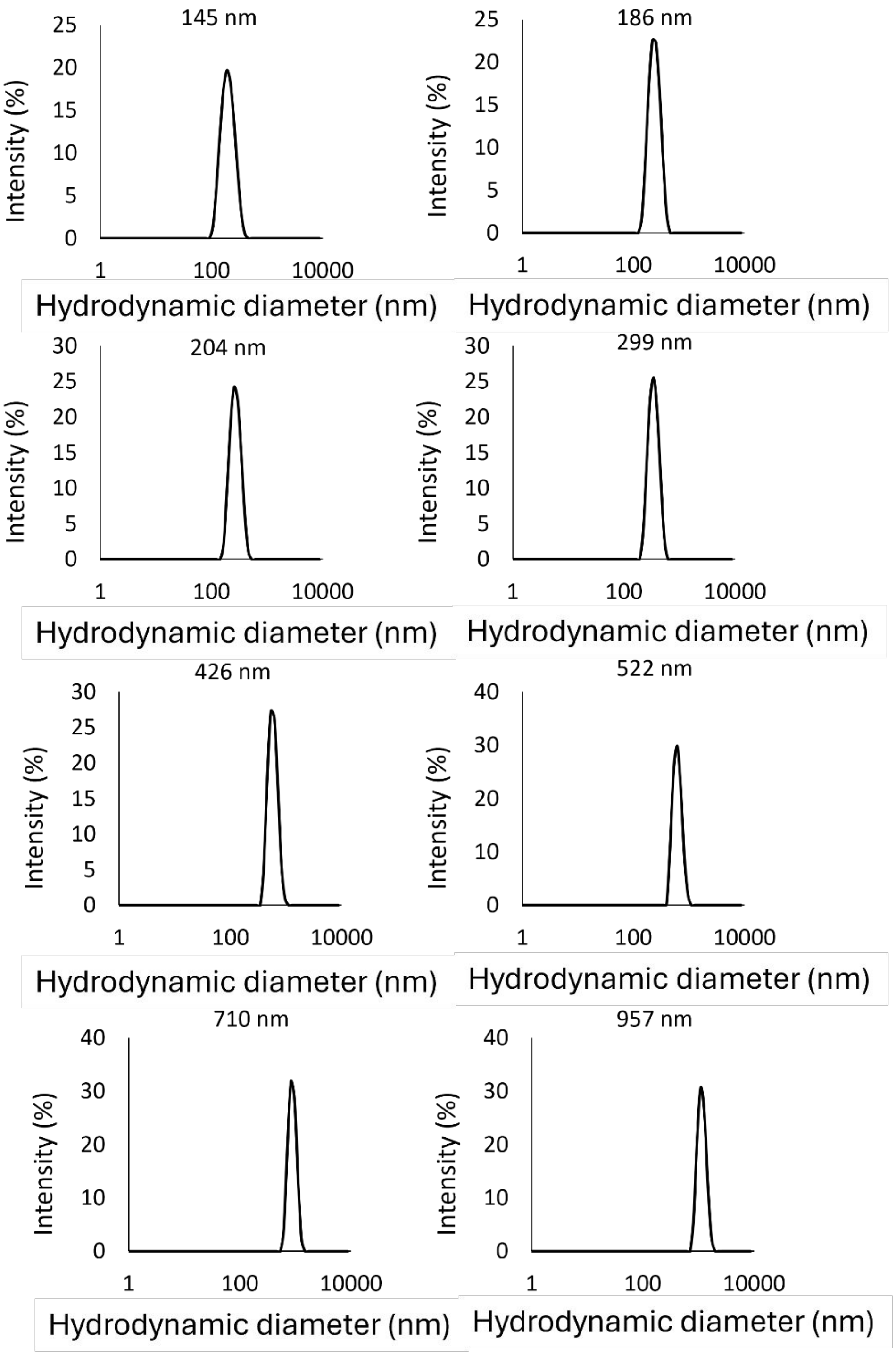

Figure S1. Dynamic light scattering (DLS) analysis for the different classes of PDNPs.

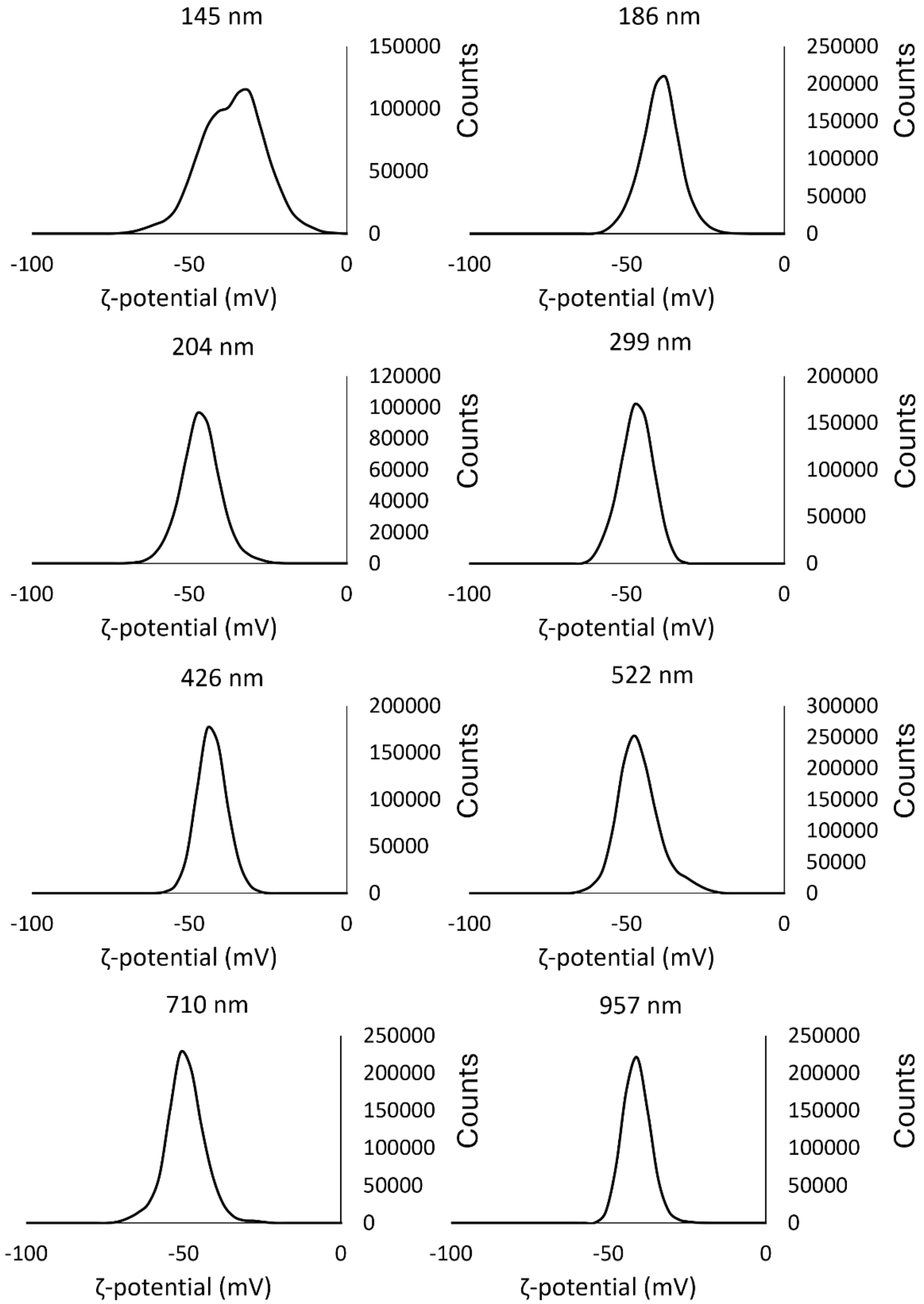

Figure S2. ζ-potential analysis for the different classes of PDNPs.

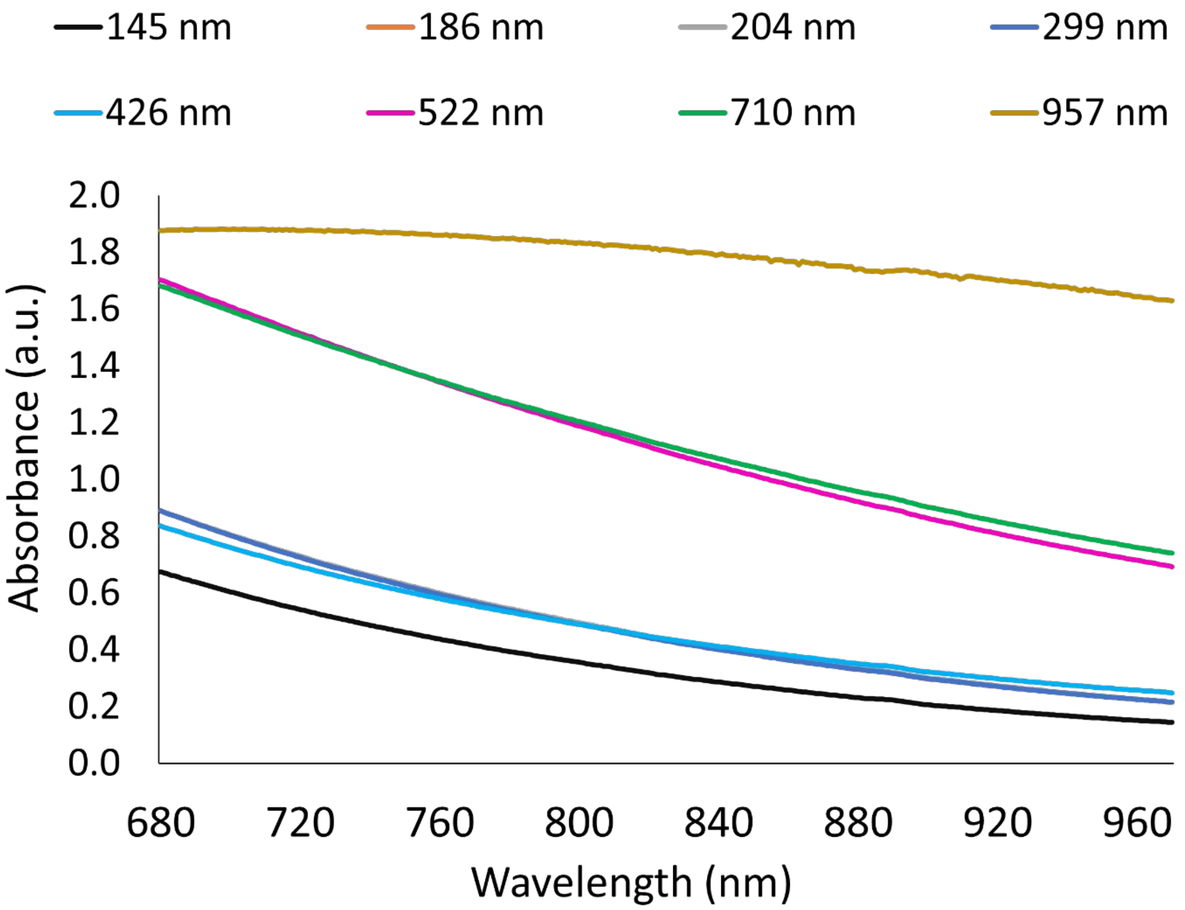

Figure S3. UV–vis–NIR absorption spectra of PDNPs with different nominal diameters, measured by spectrophotometry, showing broadband optical absorption across 680–970 nm with size-dependent intensity variations.

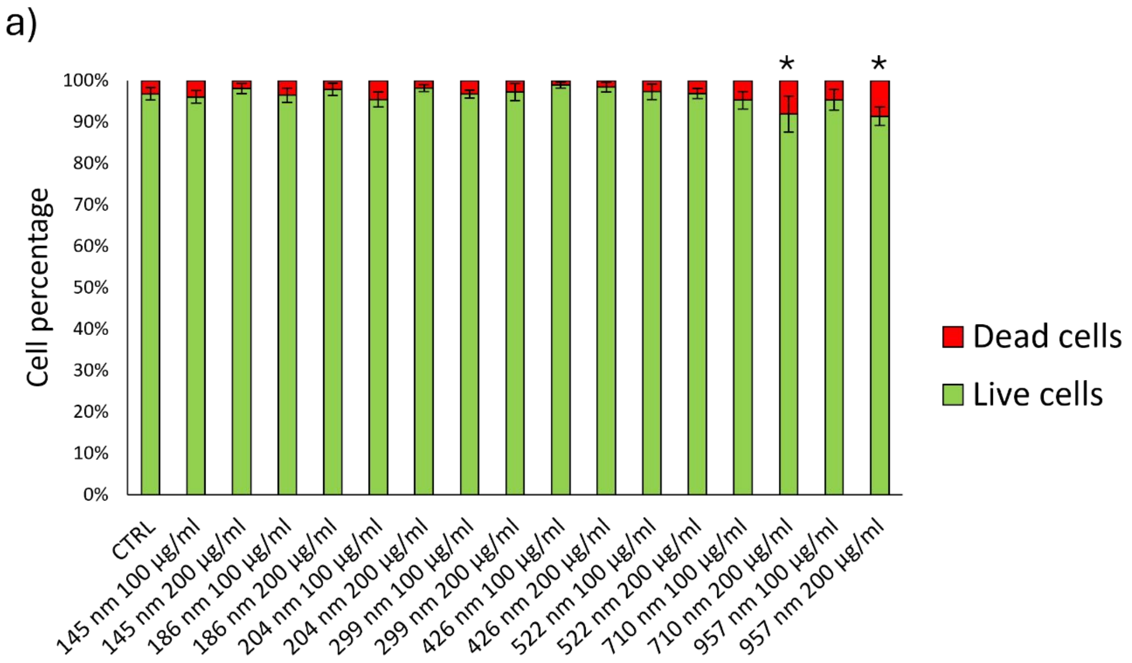

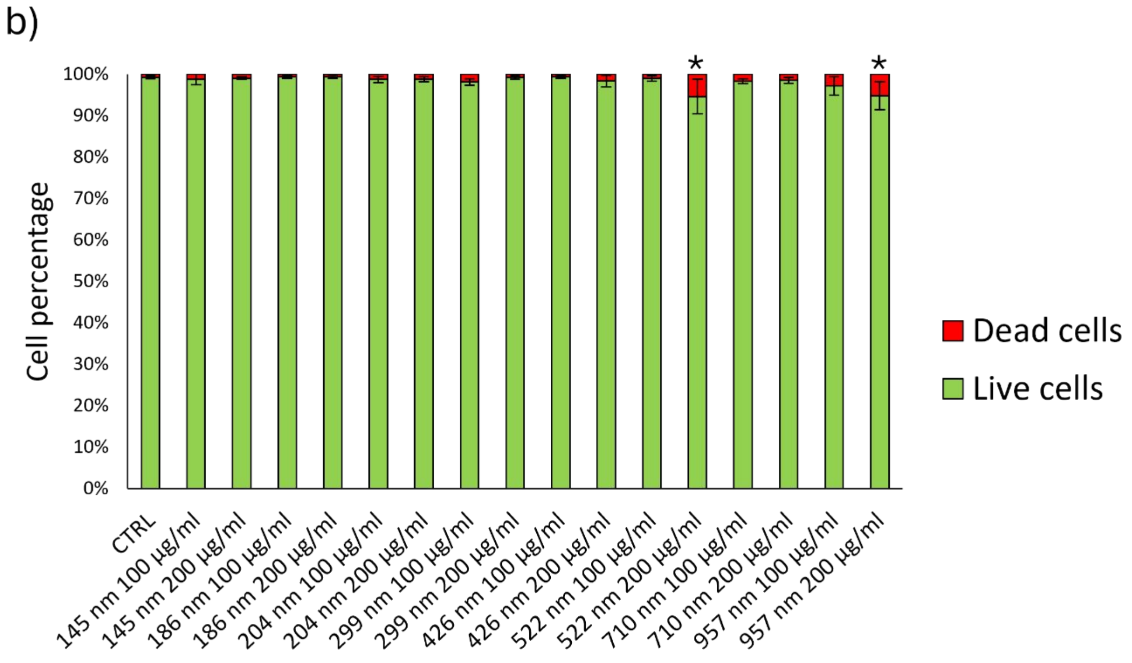

Figure S4. Quantitative evaluation of live/dead assay performed on U87 cells treated with PDNPs of various diameters and at various concentrations for a) 24 or b) 72 h.

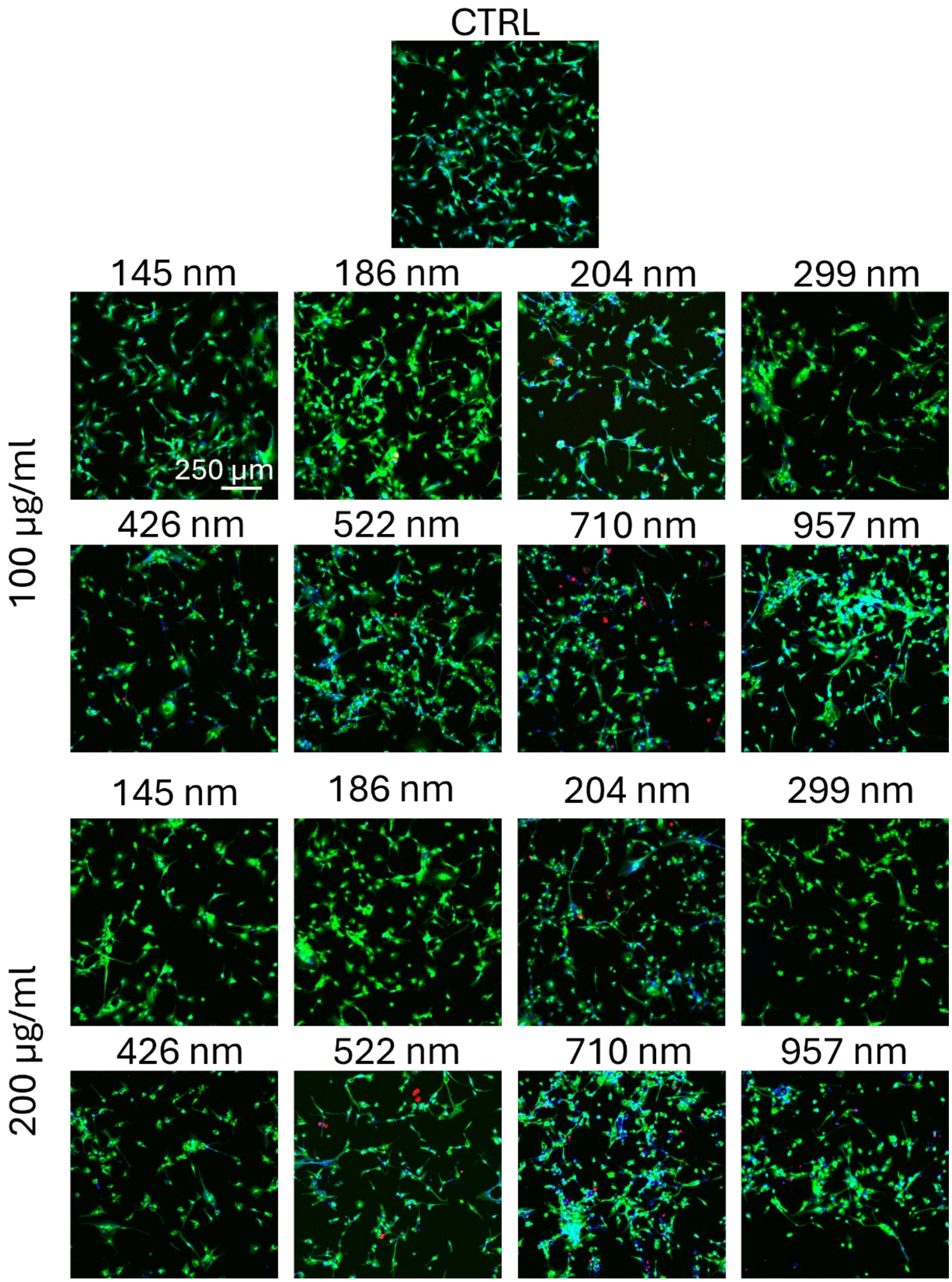

Figure S5. Representative epifluorescence images showing results of Live/Dead assay performed on U87 cells treated with PDNPs of different diameters and at different concentrations for 24 h (in green live cells, in red dead cells, in blue nuclei).

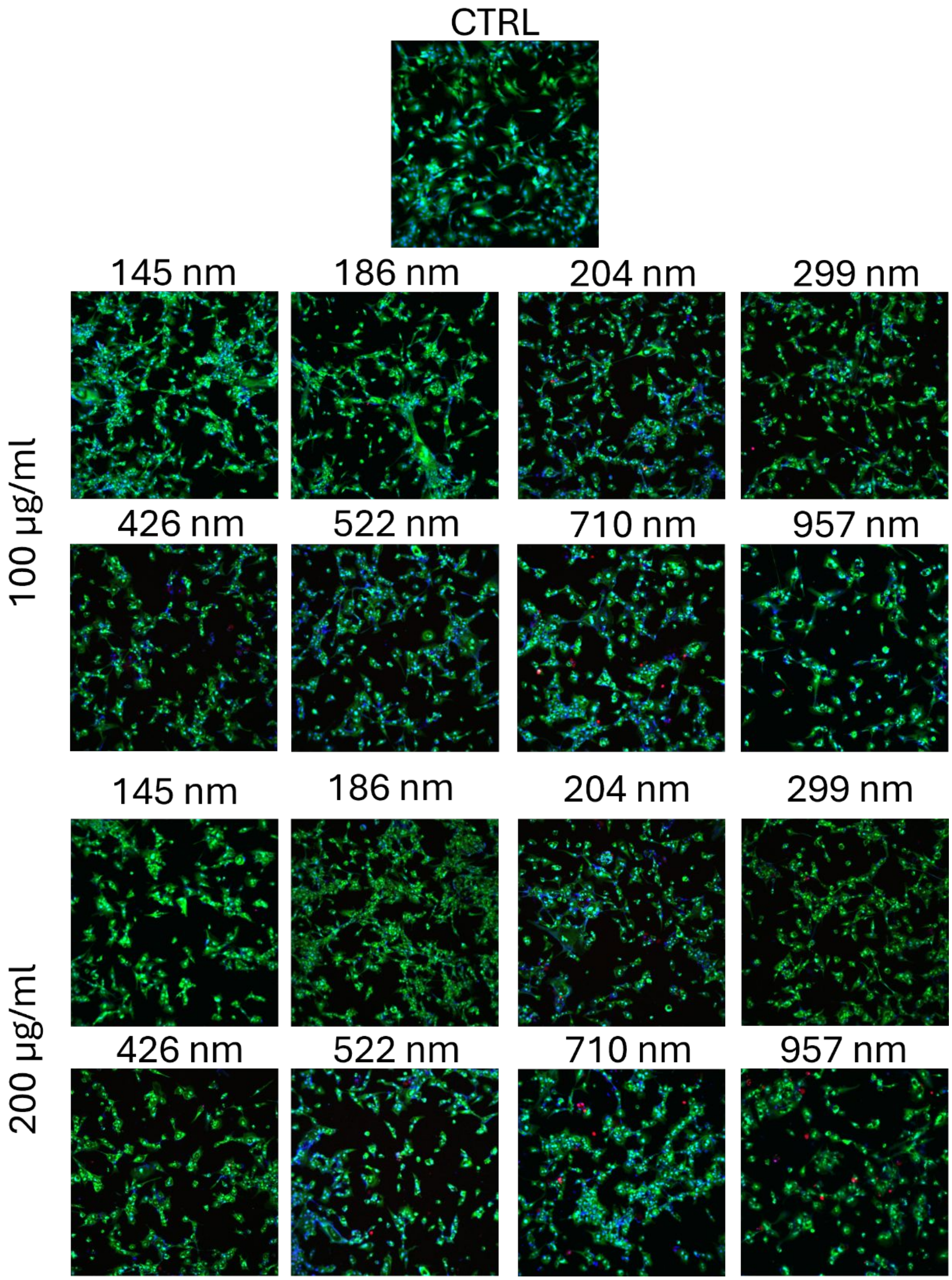

Figure S6. Representative epifluorescence images showing results of Live/Dead assay performed on U87 cells treated with PDNPs of different diameters and at different concentrations for 72 h (in green live cells, in red dead cells, in blue nuclei).

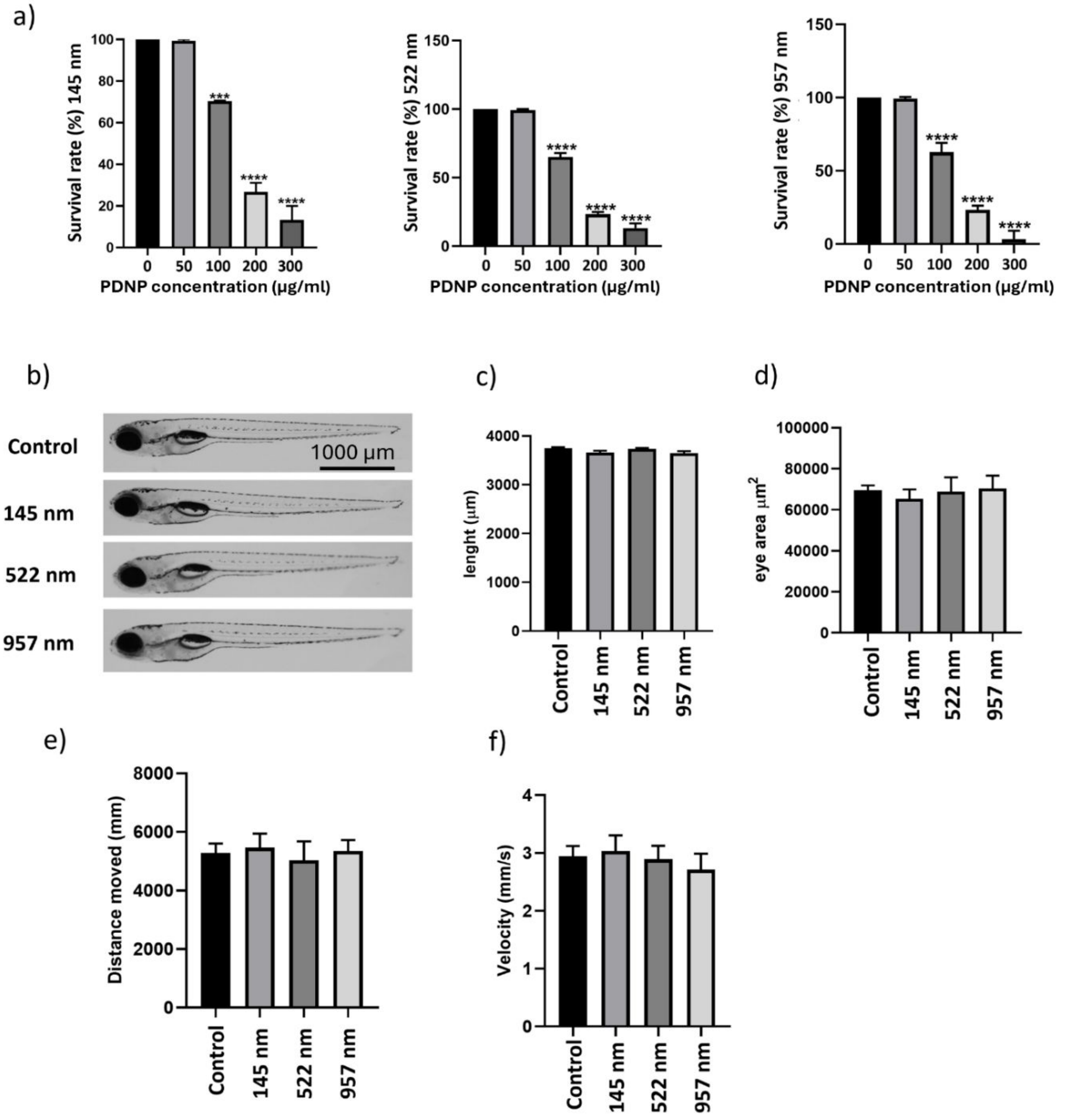

Figure S7. a) Survival rate of embryos at 72 hpf (24 h post-injection) following injection with 2 nl of nanoparticle suspensions at 50, 100, 200, and 300 µg/ml, compared to non-injected controls. b) Representative lateral images of zebrafish larvae at 120 hpf injected with 50 µg/ml nanoparticles for each size (145, 522, and 957 nm), illustrating normal morphology in lateral view. No gross developmental malformations were observed at this dose. c) Quantitative analysis of eye size in larvae injected with 50 µg/ml nanoparticles (145, 522, and 957 nm) compared to non-injected controls. No significant differences were observed between treated and control groups. d) Body length of zebrafish larvae at 120 hpf in the same groups as c), showing no significant alterations in overall growth relative to controls. Larval swimming behavior at 120 hpf measuring both e) distance moved and f) swimming velocity. No significant differences were observed smong larvae injected with 50 µg/ml nanoparticles (145, 522, and 957 nm) and non-injected controls. *** $p < 0.001$, **** $p < 0.0001$.

a)

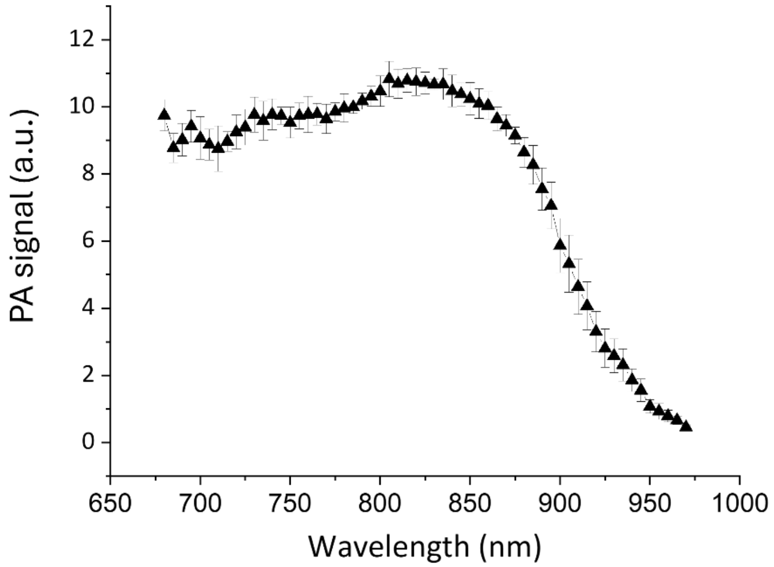

b)

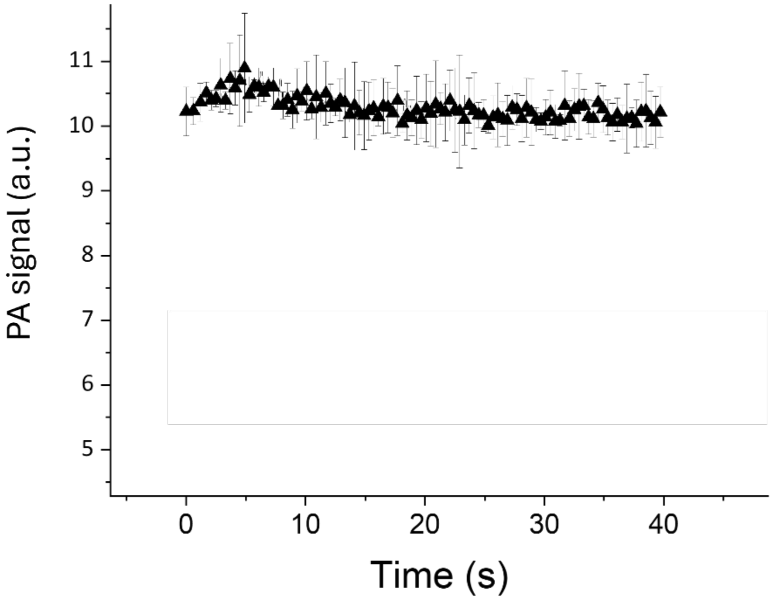

Figure S8. a) PA signal at various wavelengths and b) photostability derived from indocyanine green (ICG) at 200 μg/ml measured at 705 nm.

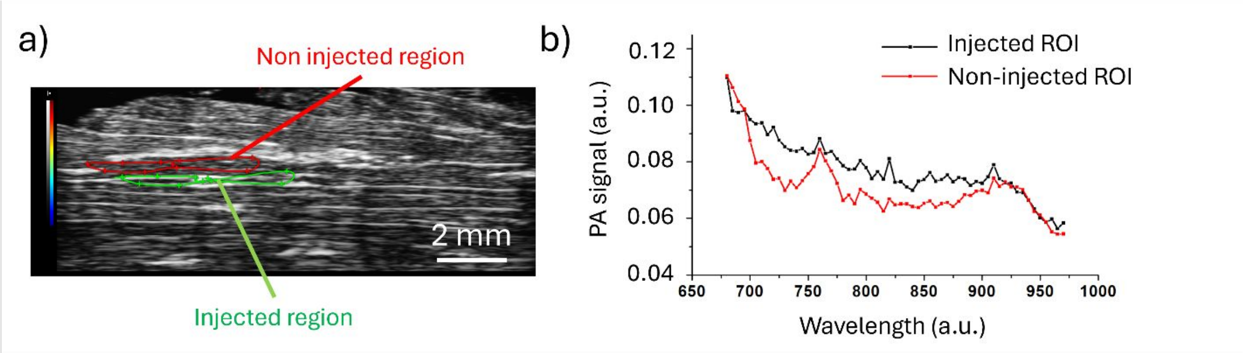

Figure S9. Control test: a) PA image of chicken breast tissue injected with PBS and b) corresponding PA signal analysis.

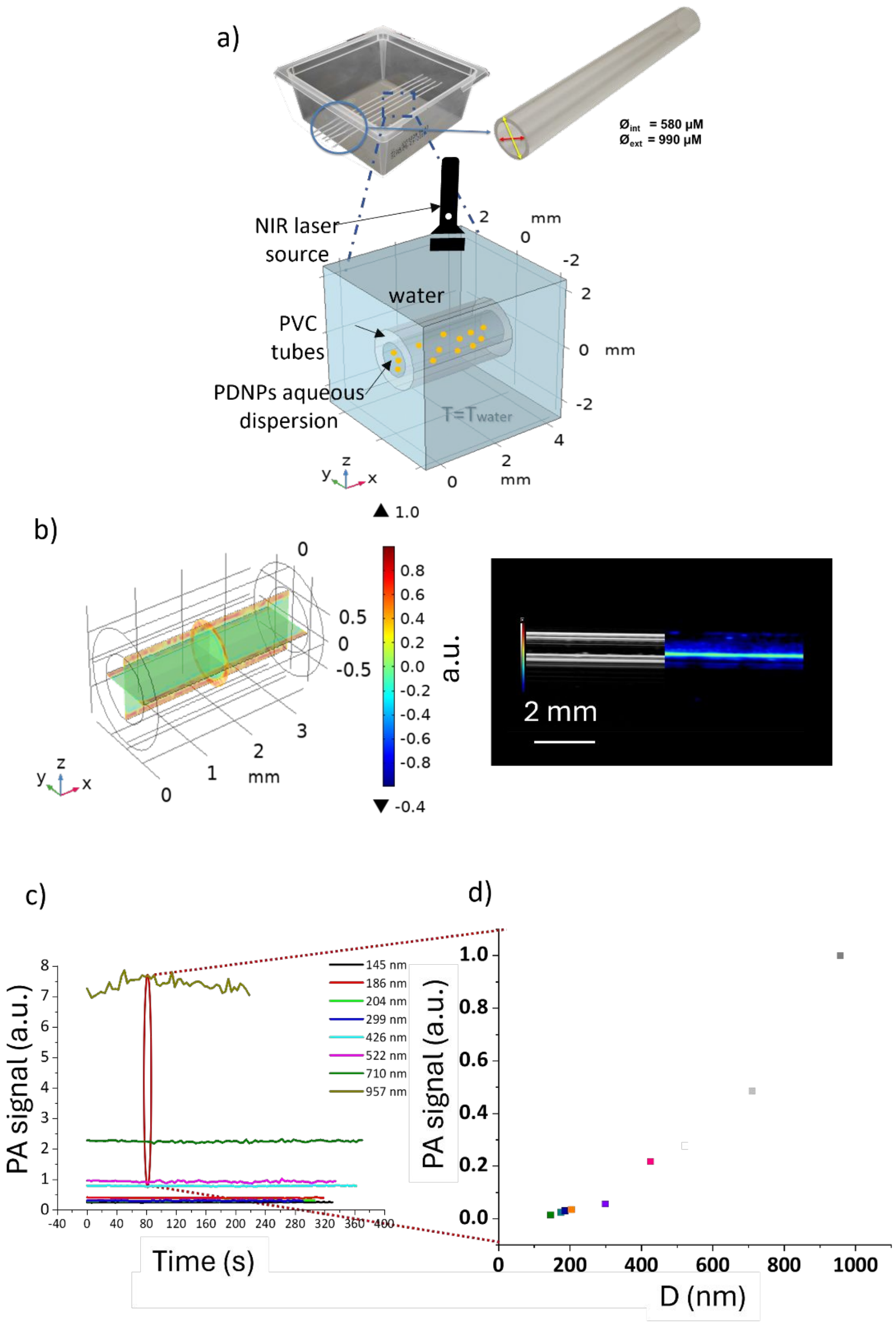

Figure S10. Modeling of the PA signal generated by an aqueous dispersion of PDNPs. a) The experimental set-up (CAD design) and the geometrical model in COMSOL. b) Normalized PA signal (a.u.) in PVC tubes generated by an aqueous dispersion of PDNPs (957 nm in nominal diameter) irradiated by a near infra-red (NIR) laser source at 705 nm: simulation results on tube cross-section (left) and experimental results (right). c) Experimental results of normalized PA signal generated by aqueous dispersions of PDNPs of different nominal diameters during prolonged laser illumination at 705 nm. d) Simulation results of normalized PA signal as a function of the PDNP nominal diameter.

**Time / PDNP nominal diameter**	145 nm	186 nm	200 nm	299 nm	426 nm	522 nm	710 nm	952 nm
1 h	237 ± 3 nm	254 ± 6 nm	362 ± 7 nm	373 ± 6 nm	464 ± 1 nm	587 ± 27 nm	700 ± 1 nm	966 ± 48 nm
24 h	236 ± 3 nm	254 ± 3 nm	362 ± 7 nm	380 ± 1 nm	470 ± 7 nm	566 ± 3 nm	740 ± 28 nm	1042 ± 18 nm
48 h	234 ± 10 nm	247 ± 4 nm	360 ± 3 nm	395 ± 4 nm	470 ± 10 nm	656 ± 15 nm	745 ± 10 nm	1064 ± 78 nm

Table S1. Average hydrodynamic diameter of PDNPs of various diameters after incubation with full cell culture medium at different time points.

**Time / PDNP nominal diameter**	145 nm	186 nm	200 nm	299 nm	426 nm	522 nm	710 nm	952 nm
1 h	0.07 ± 0.02	0.07 ± 0.02	0.06 ± 002	0.14 ± 0.04	0.07 ± 0.05	0.09 ± 0.07	0.19 ± 0.09	0.16 ± 0.04
24 h	0.08 ± 0.02	0.06 ± 0.05	0.14 ± 0.02	0.18 ± 0.03	0.12 ± 0.03	0.21 ± 0.02	0.22 ± 0.01	0.21 ± 0.08
48 h	0.06 ± 0.03	0.13 ± 0.05	0.07 ± 0.03	0.16 ± 0.06	0.42 ± 0.06	0.19 ± 0.21	0.19 ± 0.03	0.62 ± 0.31

Table S2. Average polydispersity index (PDI)of PDNPs of various diameters after incubation with full cell culture medium at different time points.

**PDNP nominal diameter**	**Average PA signal (a.u.)**	**St. Dev (a.u.)**	**Contrast (a.u.)**	**CNR**	**SNR**	**%CV**
145 nm	0.26	0.01	4.31	69.03	69.03	1.45
186 nm	0.41	0.01	7.28	66.18	66.18	1.51
204 nm	0.31	0.01	5.40	78.83	78.82	1.27
299 nm	0.32	0.01	5.44	28.56	28.55	3.50
426 nm	0.80	0.01	15.28	90.47	90.47	1.11
522 nm	0.94	0.03	18.20	34.90	34.90	2.87
710 nm	2.26	0.03	45.17	76.10	76.10	1.32
957 nm	7.40	0.19	150.34	37.59	37.60	2.66
ICG	10.34	0.33	62.63	30.19	30.50	3.27

Table S3. Average PA signal, contrast, CNR, SNR, and %CV of PDNPs of different nominal diameters (PAI was performed at 705 nm) and ICG.
